# Comprehensive analysis of the mitochondrial genome of *Rehmannia glutinosa*: insights into repeat-mediated recombinations and RNA editing-induced stop codon acquisition

**DOI:** 10.3389/fpls.2024.1326387

**Published:** 2024-05-14

**Authors:** Tiexin Zeng, Yang Ni, Jingling Li, Haimei Chen, Qianqi Lu, Mei Jiang, Lijia Xu, Chang Liu, Peigen Xiao

**Affiliations:** Institute of Medicinal Plant Development, Chinese Academy of Medical Sciences & Peking Union Medical College, Beijing, China

**Keywords:** mitochondrial genome, repeat-mediated recombination, mitochondrial plastid DNAs, RNA editing, homologous recombination

## Abstract

*Rehmannia glutinosa* is an economically significant medicinal plant. Yet, the structure and sequence of its mitochondrial genome has not been published, which plays a crucial role in evolutionary analysis and regulating respiratory-related macromolecule synthesis. In this study, the *R. glutinosa* mitogenome was sequenced employing a combination of Illumina short reads and Nanopore long reads, with subsequent assembly using a hybrid strategy. We found that the predominant configuration of the *R. glutinosa* mitogenome comprises two circular chromosomes. The primary structure of the mitogenome encompasses two mitochondrial chromosomes corresponding to the two major configurations, Mac1-1 and Mac1-2. The *R. glutinosa* mitogenome encoded an angiosperm-typical set of 24 core genes, nine variable genes, three rRNA genes, and 15 tRNA genes. A phylogenetic analysis using the 16 shared protein-coding genes (PCG) yielded a tree consistent with the phylogeny of Lamiales species and two outgroup taxa. Mapping RNA-seq data to the coding sequences (CDS) of the PCGs revealed 507 C-to-U RNA editing sites across 31 PCGs of the *R. glutinosa* mitogenome. Furthermore, one start codon (nad4L) and two stop codons (rpl10 and atp6) were identified as products of RNA editing events in the *R. glutinosa* mitogenome.

## 1 Introduction

*Rehmannia glutinosa* (Gaertn.) DC. (http://www.theplantlist.org/), a member of the Scrophulariaceae family has been widely used in traditional Chinese medicine (TCM) and is commonly known as “DiHuang” in China (Li et al., 2022). With a medicinal history spanning over two millennia, *R. glutinosa* is a vital industrial crop first documented in Shennong’s Classic of Materia Medica (Qin and Han Dynasties, 100 BC) (Li et al., 2022). The plant is processed into various forms, including fresh rehmannia root (Xian DiHuang), rehmannia dried rhizome (Sheng DiHuang), and prepared rehmannia root (Shu DiHuang) (Meng et al., 2017; Li et al., 2022). In its fresh form, *R. glutinosa* root possesses many therapeutic benefits, including antipyretic, salivary secretion enhancement, hematothermal regulation, anti-coagulative, detoxifying, and analgesic properties. It is commonly employed in the treatment of various medical conditions such as fevers, yin imbalances, glossal abnormalities, polydipsia, cutaneous eruptions, hematemesis, epistaxis, and pharyngitis (Liu et al., 2017; Meng et al., 2017; Li et al., 2022). Moreover, the Liuwei Dihuang Pill, a quintessential formulation in Traditional Chinese Medicine (TCM), features *R. glutinosa* as its principal component, demonstrating substantial efficacy in ameliorating diabetes and its associated complications (Zheng et al., 2020; Chen et al., 2021; Lu et al., 2022). *R. glutinosa* holds significant research and development value due to its extensive medicinal history and efficacy. However, cultivation is often challenged by root rot and high-stress resistance (Kim et al., 2020; Wang R et al., 2018). Traditional artificial domestication is time-consuming, and the ability of direct introduction of superior wild variety genes is limited (Fernie and Yang, 2019). Next-generation sequencing technology has enabled the integration of bioinformatics with genetic engineering, offering new possibilities for breeding *R. glutinosa* (Koenig et al., 2013; Meyer and Purugganan, 2013).

Mitochondria, biomacromolecules as essential cellular organelles, play a critical role in various metabolic processes, including the tricarboxylic acid (TCA) cycle, urea cycle, heme biosynthesis, calcium homeostasis, iron/sulfur cluster formation, gluconeogenesis, amino acid metabolism, and apoptosis (Osellame et al., 2012). Moreover, mitochondria are involved in synthesizing and folding essential biological macromolecules such as proteins, lipids, and nucleic acids, which are fundamental components of cellular structures and processes (Blomain and McMahon, 2012). Contrasting nuclear DNA (nDNA), mitochondrial DNA (mtDNA) is more susceptible to exogenous and endogenous stress due to its proximity to oxidative phosphorylation sites and the absence of protective histones in mitochondria. Although nucleoid structures offer some protection, mtDNA damage frequently occurs within mitochondria (Liao et al., 2022; Palozzi et al., 2022). The current research has demonstrated that the incidence of mtDNA damage in cells significantly surpasses that of nDNA damage (Liao et al., 2022; Palozzi et al., 2022; Roy et al., 2022). The mtDNA damage and repair mechanisms including Non-homologous end joining (NEHJ) often lead to homologous recombination in mitochondrial genomes, potentially mediated by repeat sequences (Dahal et al., 2018; Chevigny et al., 2020). Damage to mitochondria can also result in delusions, which are implicated in cytoplasmic male sterility (CMS) (Hu et al., 2014). Several CMS-related genes have been identified and characterized across various species, such as RT98-CMS rice and RT102-CMS rice (Igarashi et al., 2013; Okazaki et al., 2013). Given the central role of mitochondria in synthesizing and maintaining the presence of biological macromolecules, investigating the mitochondria of industrial crops holds substantial importance for cultivating high-quality crops. Genome research on *R. glutinosa* may yield valuable insights into the relationship between mitochondria and macromolecules, furthering our understanding of these complex interactions and their implications for crop improvement.

RNA editing events are prevalent in mitochondrial genomes and have far-reaching implications for protein function. These events often result in alterations to the amino acids specified by the genomic sequence. Such modifications not only enhance the conservation of the overall amino acid sequence but also affect the physicochemical attributes of the protein, even influencing its folding dynamics (Takenaka et al., 2008; Small et al., 2020; Kang et al., 2021). These observations underscore the pivotal role of RNA editing sites in maintaining the proper functionality of proteins. Additionally, RNA editing events appear to be intricately linked with the mechanisms of natural selection. Some researchers (Chateigner-Boutin and Small, 2010; Ichinose and Sugita, 2017) have investigated RNA editing events within the mitochondria of 17 angiosperm species. Remarkably, the nonsynonymous editing sites exhibit high conservation across these species, with approximately 80% conservation observed.

Additionally, the efficiency of the editing process is notably high, achieving an editing extent of around 80% across all examined plant species. This high level of conservation and efficiency suggests a crucial functional role for these editing events in plant mitochondrial biology. After reverse transcription into cDNA, some edited transcripts integrate into the genome through homologous recombination and are subsequently preserved. Most RNA editing sites in plant mitochondria are predominantly at the second codon position. The most frequent form of editing involves the conversion of cytosine (C) to uracil (U). This specific nucleotide alteration is thought to be correlated with an overall increase in the hydrophobicity of the resultant protein. Approximately 55% of amino acid substitutions resulting from RNA editing events exhibit a transition from hydrophilic to hydrophobic properties. This trend suggests a substantive impact on the edited protein’s physicochemical characteristics, potentially affecting its function and interaction within cellular environments (Sun et al., 2016; Mohammed et al., 2022). Additionally, the premature emergence of stop codons caused by RNA editing may result from erroneous editing, leading to the premature termination of gene translation, reducing the amino acid sequence of the encoded protein, and affecting protein function.

We successfully assembled and characterized the mitochondrial genome of *R. glutinosa*’s dual mitochondrial chromosomes in the present study. We validated its secondary structure through the lens of homologous recombination mediated by direct repeats. Additionally, we identified 507 RNA editing sites within the protein-coding regions, all of which involved the conversion of cytosine (C) to uracil (U). Our analysis also revealed the presence of two modified stop codons in the CDs of *rps10* and *atp6* and one altered start codon of *nad4L*, resulting from RNA editing events. These findings offer novel insights into the complexity and functional implications of RNA editing in the mitochondrial genome of *R. glutinosa*.

## 2 Materials and methods

### 2.1 Plant materials, DNA and RNA extraction, and sequencing

Fresh leaves of *R. glutinosa* plants (IMPLAD Accession Number: 202205002) were harvested at the Institute of Medicinal Plant Development (IMPLAD, Longitude: 116.267500° E, Latitude: 40.033056° N). After cleansing with deionized distilled water (ddH2O), the specimens were cryopreserved at −80°C. The leaf samples were partitioned into two sets designated for DNA sequencing (DNA-seq) and RNA sequencing (RNA-seq).

Genomic DNA was isolated employing the Magnetic Plant Genomic DNA Kit (Catalog No. DP342; Tiangen, China). Total RNA was extracted utilizing the RNAprep Pure Plant Plus Kit (Catalog No. DP441; Tiangen, China). For RNA-seq analysis, mRNA was selectively enriched from the total RNA pool using targeted probes to remove ribosomal RNA (rRNA). Fragmentation was executed using divalent cations in a high-temperature environment provided by the First Strand Synthesis Reaction Buffer (5X). Subsequently, after the adenylation of the 3’ ends of DNA fragments, NEBNext Adaptors featuring a hairpin loop structure were ligated, setting the stage for subsequent hybridization. The cDNA fragments with a predominant length range of 370 to 420 base pairs were isolated to construct the fragmented library via the AMPure XP system (Beckman Coulter, Beverly, USA). The sequencing library was constructed using the TIANSeq Fast DNA Library Kit (Illumina; Catalog No. NG102), and sequencing was performed on an Illumina NovaSeq 6000 platform (Illumina, Inc.; San Diego, CA, USA).

For Oxford Nanopore sequencing, high molecular weight (HMW) DNA was isolated using the NEB Monarch HMW DNA Extraction Kit (Catalog No. T3060L; New England Biolabs, England). Mechanical shearing of the genomic DNA to an average fragment size of approximately 10 kb was accomplished using the Covaris g-TUBE (Thermo Fisher, USA). The DNA library was assembled using the DNA Library Kit (Catalog No. SQK-LSK110) and sequenced on a PromethION platform (Novogene Co., Ltd., Beijing, China).

### 2.2 Genome assembly and annotation

Illumina short-read sequences were processed using Trimmomatic software, employing the default settings (Bolger et al., 2014). Nanopore long-read sequences were filtered using Guppy software, also with default settings (Wick 2017). A hybrid assembly approach was implemented for the assembly of organelle genomes. For the assembly of the plastid genome (plastome), we utilized GetOrganelle software (Jin et al., 2020) to extract plastid-specific reads from the Illumina dataset, applying parameters “-R 15 -k 21,45,65,85,105 -F embplant_pt”. These reads were assembled into a unitig graph, and bifurcation structures corresponding to inverted repeat regions were resolved by aligning the Nanopore reads to these structures via the Unicycler software (Wick et al., 2017). The orientation of the resulting assembled genome was subsequently refined using Novowrap (Wu et al., 2021). For the mitochondrial genome (mitogenome), a similar hybrid assembly strategy was employed. Initially, GetOrganelle was used to isolate mitochondrial reads from the raw data, employing parameters “-R 50 -k 21,45,65,85,105 -P 1000000 -F embplant_mt”. These reads were then assembled into a unitig graph, and bifurcation structures were resolved through nanopore read alignment via Unicycler software (Wick et al., 2017).

Annotation of the plastome was performed using both CPGAVAS2 (Shi et al., 2019) and CPGView (Liu et al., 2023). Protein-coding genes (PCGs) and ribosomal RNA (rRNA) sequences within the mitogenome were annotated using Geseq (Tillich et al., 2017). Transfer RNA (tRNA) molecules were identified using tRNA-scan (Lowe and Eddy, 1997), version 1.4. A graphical representation of the mitogenome was generated using OGdraw (Greiner et al., 2019). All organelle genome annotations underwent meticulous review and were manually corrected as needed using the Apollo software suite (Misra and Harris, 2006).

### 2.3 Repeat elements, mitochondrial plastid DNAs, and mitochondrial nuclear DNAs analysis

Microsatellite sequence repeats (SSRs) were detected using the MISA tool with parameters specified as “1-10 2-6 3-5 4-5 5-5 6-5.” Tandem repeats were ascertained using the Tandem Repeats Finder (TRF) with parameters set at ‘2 7 7 80 10 50 500 -f -d -m’.

Mitochondrial Plastid Sequences (MTPTs) were discerned through a reciprocal comparison strategy, employing BLASTn (version 2.2.30+) with its default parameters. The plastid genome (plastome) was assembled utilizing Illumina sequence reads via the GetOrganelle software. Comparative analysis between the plastome and the mitochondrial genome (mitogenome) was performed using BLASTn, employing specific parameters: e-value set to 1e-6 and word size configured at 7 (Chen et al., 2015). BLASTn hits shorter than 100 base pairs were excluded from the analysis. Subsequently, MTPT gene clusters within the mitogenome were delineated and defined as contiguous gene assemblies in the plastome devoid of intervening mitochondrial genes. These MTPT gene clusters were visually represented in a circular map generated using TBtools (version 1.076).

To identify putative Nuclear Mitochondrial DNA segments (NUMTs), the nuclear genome of R. glutinosa (GenBank accession JABTTQ000000000.1) was compared against the mitogenome using BLASTn. Specific BLASTn parameters were as follows: e-value of 1e-5, word size of 9, gap opening cost of 5, gap extension cost of 2, match reward of 2, mismatch penalty of -3, and turning off the dust filter. The BLASTn output was visualized using TBtools (Chen et al., 2020). Segments identified as potential NUMTs were further annotated using GeSeq software. Additionally, the nuclear genomes of R. glutinosa were similarly probed for putative NUMTs.

### 2.4 Identification and validation of repeat mediated recombination

To investigate the influence of repeat sequences on both intermolecular and intramolecular recombination events within the mitochondrial genome of R. glutinosa, we employed BLASTn analysis. The search parameters were meticulously chosen, incorporating an Expectation value (E-value) threshold of 1E-6 and a word size setting of 7 to identify relevant repeat sequences rigorously (Chen et al., 2015). Sequence segments of 500 base pairs (bp) in length surrounding the repeats were extracted to assess potential recombination products near the repeats based on anticipated sequences preceding and succeeding recombination. Subsequently, Nanopore long reads were mapped to the extracted sequence segments of the four configurations, and the repeat-spanning reads were enumerated.

To investigate putative recombination products identified through mapping PacBio long reads, polymerase chain reaction (PCR) primers were designed at the junction of repetitive sequences and recombination fragments using the Primer 3 web service (Untergasser et al., 2012). PCR reactions were performed in 50 μL volumes, consisting of 23 μL water, 25 μL 2 × Taq PCR Master Mix, 1 μL of each primer, and 1 μL DNA. The reactions were performed on a Pro-Flex PCR system (Applied Biosystems, Waltham, MA, USA). Subsequently, the PCR products were separated and visualized on 1.0% agarose gels. Finally, the PCR amplicons were sequenced using the Sanger method to confirm the recombination events.

### 2.5 Phylogenetic analysis

To construct a phylogenetic tree, we downloaded 21 Lamiales mitogenome sequences, including the original version of *R. glutinosa* (OM397952.2), from the National Center for Biotechnology Information (NCBI) database. The common genes from 21 mitochondrial genomes were extracted and concatenated using Phylosuite (Zhang D et al., 2020). Subsequently, the DNA sequences of the 16 protein-coding genes (PCGs) shared among these ten mitogenomes were extracted (**Table 1**). These sequences were aligned with MAFFT (v7.450) (Rozewicki et al., 2019), and a phylogenetic tree was constructed using Phylosuite with the maximum likelihood (ML) method based on the alignment. The credibility of the phylogenetic tree was assessed by performing bootstrap testing with 1,000 replications. Finally, the resulting maximum-likelihood tree was visualized using iTOL (https://itol.embl.de/) (Letunic and Bork, 2021).

**Table 1:** Lamiales mitogenome sequences for the construction of the phylogenetic tree.

Family	Species	NCBI Accession Number
Orobanchaceae	*Rehmannia glutinosa*	This study
	*Rehmannia glutinos*	OM397952.2
	*Rehmannia chingii*	OR601177.1
	*Aeginetia indica*	MW851294.1
	*Castilleja paramensis*	NC_031806.1
	*Christisonia kwangtungensis*	OM219025_7.1
Lamiaceae	*Salvia miltiorrhiza*	NC_023209.1
	*Rotheca serrata*	NC_049064.1
	*Pogostemon heyneanus*	MK728874.1
	*Scutellaria tsinyunensis*	MW553042.1
	*Scutellaria barbata*	NC_065025.1
	*Scutellaria franchetiana*	NC_065026.1
	*Ajuga reptans*	NC_023103.1
	*Ajuga ciliata*	MT075725_6.1
	*Vitex trifolia*	NC_065806.1
Plantaginaceae	*Aragoa cleefii*	OK514182.1
	*Aragoa abietina*	OK514181.1
Gesneriaceae	*Boea hygrometrica*	NC_016741.1
	*Haberlea rhodopensis*	MH757117.1
Lentibulariaceae	*Utricularia reniformis*	NC_034982.1
	*Genlisea tuberosa* voucher VFOM2001	OK274069.1
Oleaceae	*Ligustrum quihoui*	MN723864.1
	*Osmanthus fragrans*	NC_060346.1

### 2.6 Identification and validation of RNA editing sites

To delineate both RNA editing sites and Single Nucleotide Polymorphism (SNP) loci, we initially extracted the coding domains (CDs) of each protein-coding gene (PCG), flanked by 100 base pair (bp) regions to serve as reference sequences. To detect SNP loci, genomic DNA sequencing reads were aligned to the reference above sequences using the Burrows-Wheeler Aligner (BWA; version 0.7.12-r1039) (Li and Durbin, 2010), with all parameters set to default. SNP loci were subsequently identified using REDItools (version 2.0), adopting identical parameters for RNA editing site identification: a minimum coverage of 5 reads and a frequency threshold of ≥ 0.1. Following this, RNA editing sites were ascertained utilizing REDItools (version 2.0) (Picardi and Pesole, 2013), with the criteria set at a coverage threshold of ≥ 5 reads and a frequency threshold of ≥ 0.1 (Wu et al., 2017). The resultant mapping data, specifically at the RNA editing loci with a minor variant frequency of ≥ 0.1, were visualized via the Integrative Genomics Viewer (IGV; version 2.15.1) (Milne et al., 2010).

## 3 Results

### 3.1 General feature of the *R. glutinosa* mitochondrial genome

**Figure 1:**
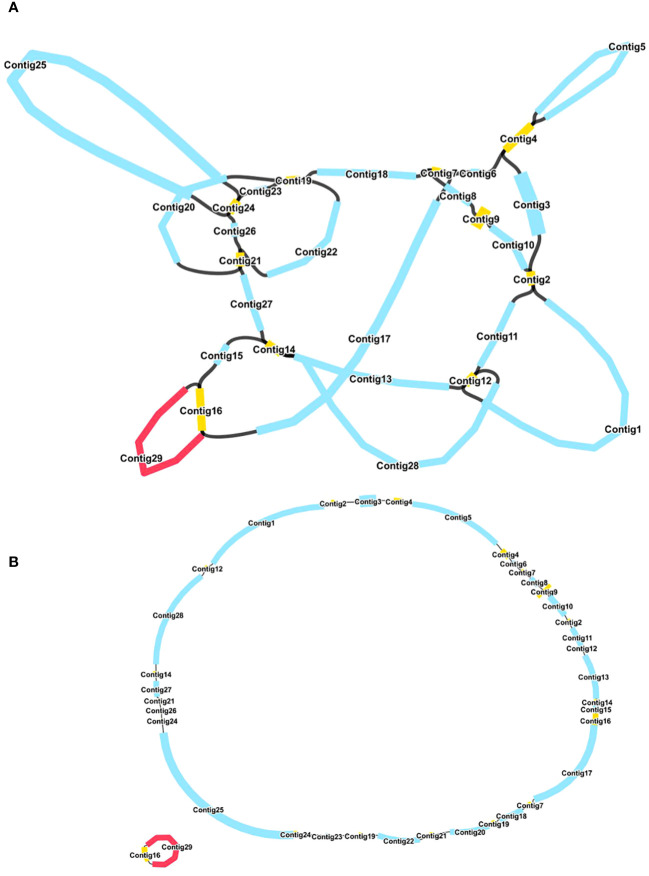
A schematic representation of the assembly process for the *R. glutinosa* mitogenome is provided. **(A)** A unitig graph for the *R. glutinosa* mitogenome was generated through *de novo* assembly of Illumina reads using Unicycler. This unitig graph consisted of seven contigs (depicted in yellow) that formed double bifurcating structures (DBSs). Each DBS exhibited two secondary configurations based on the Nanopore long reads. **(B)** A schematic diagram of the mitochondrial chromosome 1 (MC1, represented by a blue circle) and mitochondrial chromosome 2 (MC2, represented by a red circle) of *R. glutinosa* following the resolution of DBSs using long reads is presented. The contigs illustrated in blue and red correspond to chromosomes 1 and 2, respectively.

The mitochondrial genome of *R. glutinosa* represented the first published genome of the genus *Rehmannia* and the fifth mitochondrial genome within the Orobaceae family. Genomic assembly was performed using Illumina and Nanopore sequencing technologies, generating 10.2 GB and 19.8 GB reads, respectively. The coverage depth of the long and short reads mapped to the *R. glutinosa* mitogenome sequences was obtained using samtools (v1.3.1) (Li et al., 2009) (**Supplementary Figures S1, 2**). *De novo* assembly of Illumina short reads was performed using the GetOrganelle software. Repeated sequences were resolved by mapping the Nanopore long sequences. Subsequently, Unicycler software was used to extract 29 contigs to construct unitig graphs, including ten double-bifurcating structures (DBS) (**Figure 1A**). The abundance of each configuration of DBS were calculated by mapping Nanopore long reads to the reference sequences using Unicycler. These configurations were further used for final assembly, and the results of the Unicycler analysis were subsequently loaded into bandage software with the “Merge all possible nodes” module. As a result, two chromosomes of the mitotic genome of *R. glutinosa* were obtained (**Figure 1B**).

**Figure 2:**
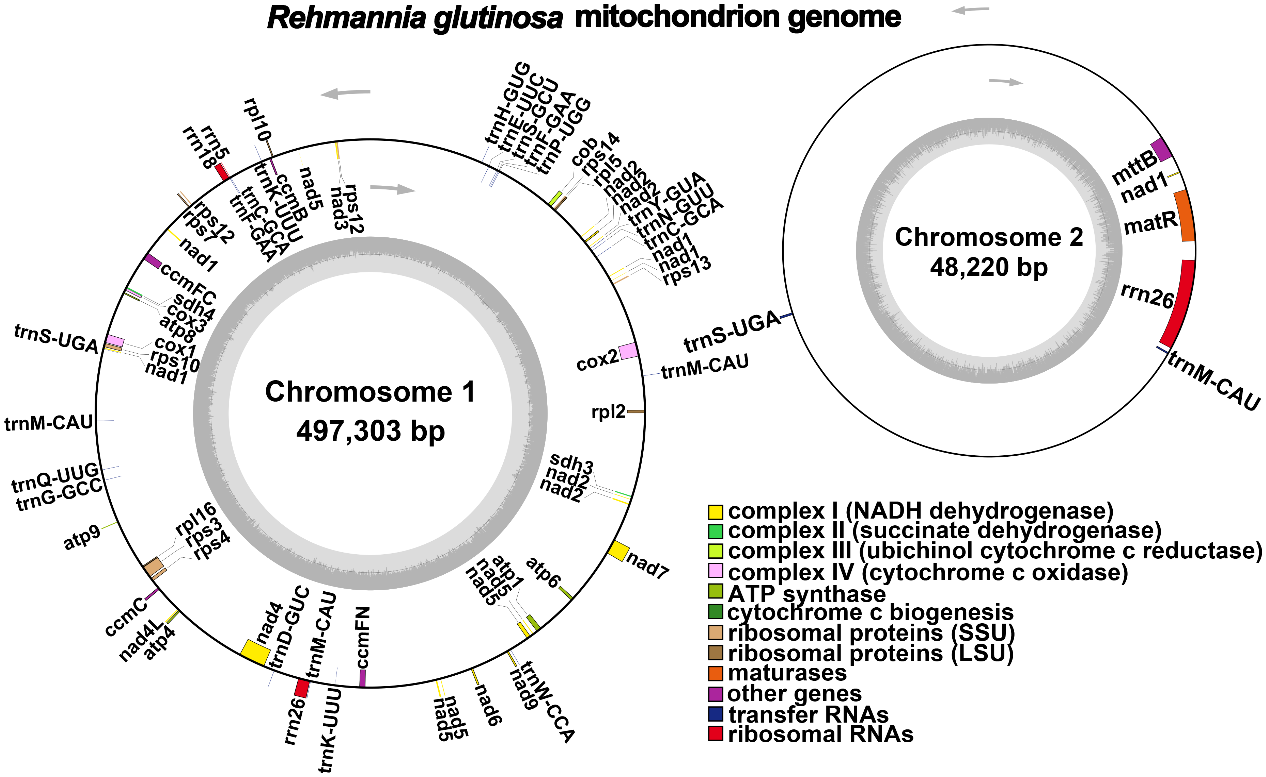
A schematic representation of the circular mitochondrial chromosome 1 and mitochondrial chromosome 2 of *Rehmannia glutinosa* is provided. Genes depicted on the inner side correspond to the negative strand, while those on the outer side represent the positive strand. Genes containing introns are marked with an asterisk (*). The gray circle illustrates the GC content, with an inner circle within the GC content graph denoting the 50% threshold. Different functional categories are indicated by the colors shown in the accompanying legend.

The *R. glutinosa* mitogenome had two chromosomes of 545,523 bp (chromosome 1 with 497,303bp, chromosome 2 with 48,220bp), and its entire GC was 45% (T 27.6%, C 22.5%, A 27.4%, G 22.5%). The GC content of *R. glutinosa* and its relative species ranged from 43.27% to 45.62%, and the genome length ranged from 225,612 bp-1,860,774 bp (**Table 2**). We annotated the mitochondrial genome, and the categorization of genes is shown in **Table 3**. The core genes consisted of five ATP synthase genes, nine NADH dehydrogenase genes, three cytochrome C biogenesis genes, three cytochrome C oxidase genes, ubiquinol cytochrome c reductase, a transport membrane protein, a maturase. The variable genes consisted of 4 large subunits of ribosome proteins (*rpl2, rpl5, rpl10*, and *rpl16*), seven small subunits of ribosome proteins (*rps3, rps4, rps7, rps10, rps12, rps13* and *rps14*), three rRNA genes (*rrn*5, *rrn*18, and *rrn*26), and two respiratory genes (*sdh3* and *sdh4*). A total of 15 unique tRNA genes were identified based on tRNAscan-SE. The schematic genome is presented in **Figure 2**.

**Table 2:** Comparative genomic analysis of Lamiales mitogenome sequences.

Species	Accession	Total Length	Average GC Content (%)	A Proportion (%)	T Proportion (%)	C Proportion (%)	G Proportion (%)
*Rehmannia glutinosa*	ON951335_6	545523	44.98343791	27.43367374	27.58288835	22.46798027	22.51545764
*Rehmannia chingii*	OR601177.1	783161	44.78325657	27.61539454	27.60134889	22.33001899	22.45323758
*Rehmannia glutinosa*	PP035761.1	545329	44.94387792	27.60792109	27.44820099	22.42206081	22.5218171
*Haberlea rhodopensis*	MH757117.1	484138	44.10374728	27.72432653	28.16944755	22.1127034	21.99104388
*Pogostemon heyneanus*	MK728874.1	380655	44.67483679	27.55303359	27.77212962	22.40979365	22.26504315
*Ligustrum quihoui*	MN723864.1	848451	44.55767039	27.56859265	27.87373696	22.23369411	22.32397628
*Ajuga ciliata*	MT075725_6.1	365414	45.34856355	27.00799641	27.64344004	22.71013152	22.63843203
*Scutellaria tsinyunensis*	MW553042.1	354073	45.26044064	27.44179873	27.29776063	22.57528815	22.6851525
*Aeginetia indica*	MW851294.1	401628	43.53979304	28.39642654	28.06378041	21.76242692	21.77736612
*Boea hygrometrica*	NC_016741.1	510519	43.27165101	28.1893524	28.53899659	21.73905379	21.53259722
*Ajuga reptans*	NC_023103.1	352069	45.09712585	27.33924316	27.56363099	22.6322113	22.46491455
*Salvia miltiorrhiza*	NC_023209.1	499236	44.38762429	27.8687835	27.74359221	22.2632182	22.12440609
*Castilleja paramensis*	NC_031806.1	495499	43.52037037	28.27997635	28.19965328	21.68581571	21.83455466
*Utricularia reniformis*	NC_034982.1	857234	43.97702378	28.03435235	27.98757399	21.99784423	21.97917955
*Rotheca serrata*	NC_049064.1	482114	45.53736253	27.37443841	27.08819906	22.83795949	22.69940305
*Osmanthus fragrans*	NC_060346.1	563202	44.58293827	27.66254381	27.75451792	22.38273302	22.20020525
*Scutellaria barbata*	NC_065025.1	372525	45.19374539	27.73209852	27.0741561	22.59955708	22.59418831
*Scutellaria franchetiana*	NC_065026.1	354302	45.28566026	27.27306084	27.44127891	22.74613183	22.53952842
*Vitex trifolia*	NC_065806.1	274779	45.62102635	27.00533884	27.37363481	22.55048603	23.07054033
*Pedicularis kansuensis*	NC_072932.1	273598	44.28979744	27.95524821	27.75495435	22.01989781	22.26989963
*Pedicularis chinensis*	NC_072955.1	225612	44.42316898	28.08760172	27.4892293	22.37248019	22.05068879
*Genlisea tuberosa voucher VFOM2001*	OK274069.1	729765	43.38848807	28.36789926	28.24320158	21.64751667	21.74097141
*Aragoa abietina*	OK514181.1	365087	44.95394248	27.58027539	27.46578213	22.60502291	22.34891957
*Aragoa cleefii*	OK514182.1	365824	44.92952895	27.61163838	27.45883266	22.56631604	22.36321291
*Christisonia kwangtungensis*	OM219025_7.1	633096	44.62545965	27.70211785	27.67242251	22.30246282	22.32299683
*Rehmannia glutinosa*	OM397952.1	554134	44.92956577	27.56282776	27.50760646	22.49997293	22.42959284
*Cistanche deserticola chromosome 1-4*	ON890398_41.1	1860774	44.55420164	27.78080519	27.65972654	22.20393234	22.3502693

**Table 3:** Gene contents in the mitogenome of *R. glutinosa*.

Group of genes	Name of genes
ATP synthase	*atp1^a^, atp4 ^a^, atp6 ^a^, atp8 ^a^, atp9 ^a^*
Cytochrome c biogenesis	*ccmB ^a^, ccmC ^a^, ccmFc ^b^, ccmFn ^a^*
Ubichinol cytochrome c reductase	*Cob ^a^*
Cytochrome c oxidase	*cox1, cox2 ^b^, cox3 ^a^*
Maturases	*matR ^a^*
Transport membrane protein	*mttB ^a^*
NADH dehydrogenase	nad1*, nad2*, nad3 ^a^, nad4 ^c^, nad4L ^a^, nad5* nad6 ^a^, nad7 ^c^, nad9 ^a^
Ribosomal protein large subunit	*rpl2 ^a^, rpl5 ^a^, rpl10 ^b^, rpl16*
Ribosomal protein small subunit	*rps3, rps4 ^a^, rps7, rps10 ^a^, rps12 ^a^, rps13 ^a^, rps14 ^a^*
Succinate dehydrogenase	*sdh3, sdh4*
Ribosomal RNA	*rrn5, rrn18, rrn26*
Transfer RNA	*trnC-GCA, trnD-GUC, trnE-UUC, trnF-GAA, trnG-GCC, trnH-GUG, trnK-UUU, trnM-CAU, trnN-GUU, trnP-UGG, trnQ-UUG, trnS-GCU, trnS-UGA, trnW-CCA, trnY-GUA*

“a”, “b”, and “c”: genes with one, two, and four exons, respectively. “*”: genes with two copies.

In this study, we analyzed the mitochondrial genome of *R. glutinosa* in this research and compared it with the publicly available genome sequence OM397952.2 (**Supplementary Figure S1**). Our findings indicate a strong collinearity between the two genomes, which is consistent from 1 bp up to 352,181 bp. Notably, there are repeated fragments spanning from 352,182 bp to 361,101 bp, and an extensive inverted repeat sequence can be observed from 419,346 bp to 547,032 bp.

### 3.2 Repeat elements analysis

**Figure 3:**
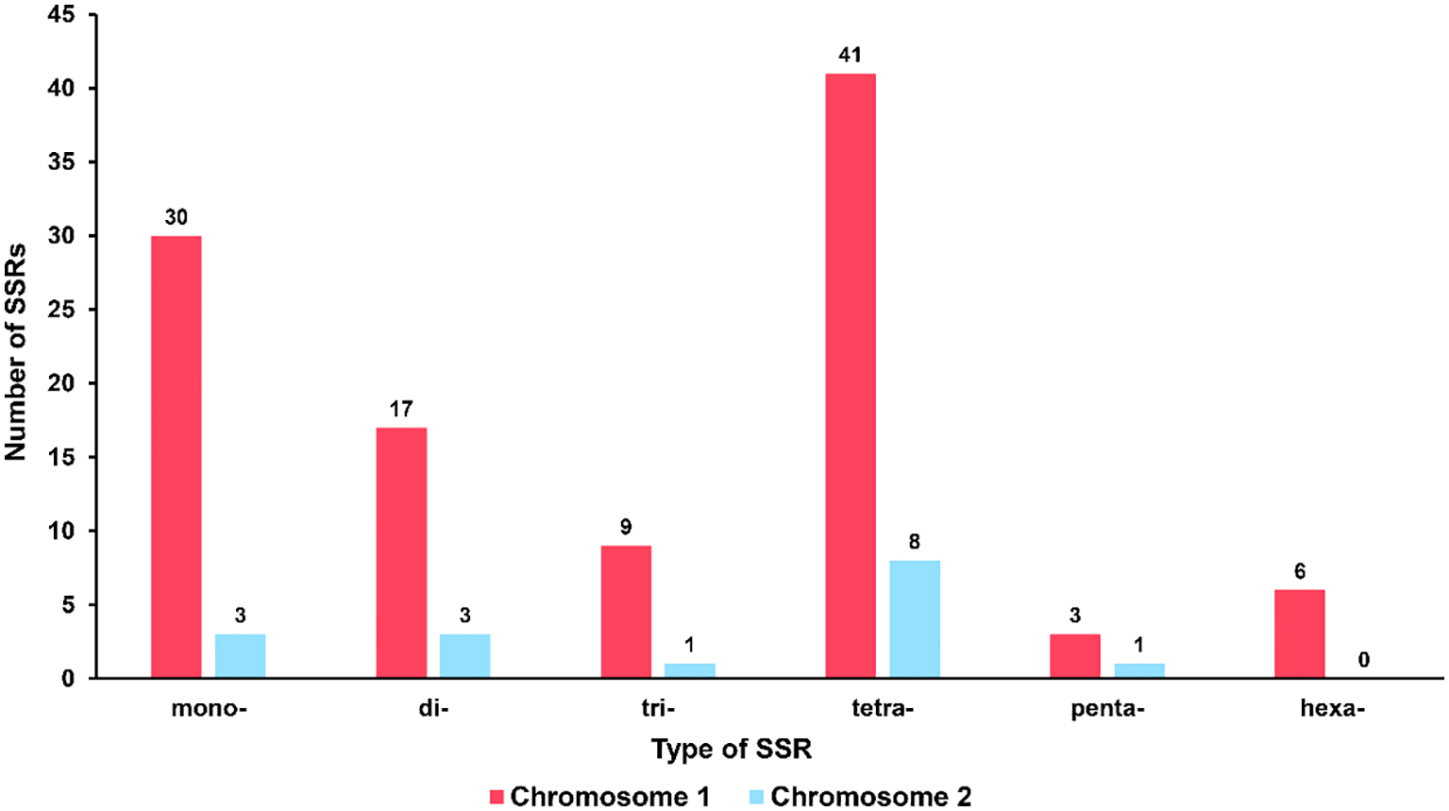
Type and quantity of SSR. The red column represents chromosome 1, and the blue column represents chromosome 2.

Microsatellites, also known as simple sequence repeats (SSRs), are short repetitive DNA units composed of mononucleotide, dinucleotide, trinucleotide, tetranucleotide, or pentanucleotide motifs that are predominantly present in eukaryotic genomes [14]. In the mitochondrial genome of *R. glutinosa*, 100 and 16 SSR markers were identified in the major and secondary chromosomal molecules, respectively (refer to **Figure 3**; **Supplementary Tables S2**, **3**). All six types of SSRs were detected in the mitochondrial genome, with 30, 17, 9, 41, and 3 SSRs having mono-, di-, tri-, tetra-, penta- or hexanucleotide repeat units in the major chromosomal molecule, and 3, 3, 1, 8, and 1 SSRs having mono-, di-, tri-, tetra-, or pentanucleotide repeat units in the chromosome 2, respectively. The most commonly occurring SSRs in the mitochondrial genome of *R. glutinosa* had a four-nucleotide repeat unit, accounting for 42.2% of all repeats. These microsatellite markers have the potential to serve as identification markers of *R. glutinosa*.

Tandemly repeated DNA sequences are characterized by a unit length greater than six base pairs and are highly variable components of the genome [15]. These repeats are commonly found in intergenic regions, although some can be located within coding sequences or pseudogenes. Six tandem repeat sequences were detected within chromosome 1 of the *R. glutinosa* mitogenome, with lengths ranging from 14 to 23 base pairs (**Supplementary Tables S3**).

### 3.3 Recombination mediated by repeat sequences

The mitochondrial genome of plants could not be fully represented by a single cyclic molecule, as rearrangement mediated by repeated sequences may occur to varying degrees. To investigate the possible homologous recombination in the mitochondrial genome of *R. glutinosa*, we detected 87 pairs of repetitive sequences in the mitochondrial genome of *R. glutinosa* using BLASTN with 1E-5. Based on Nanopore long reads, we carefully examined each pair of repetitive sequences for their support with long reads, and found that three pairs might support homologous recombination (**Table 4**; **Supplementary Table S4**). The length of these repeats is between 2,795 and 7,933 bp.

**Table 4:** The details of three direct repeats.

The ID of the HSP	Identity between the repeat units (%)	Alignment Length	Mismatches	Gap Openings	Repeat unit 1		Repeat unit 2		E-value	Type	Number of Long Reads Mapped to Configuration		Percentage of Minor Configuration
					start	end	start	end			Major	Minor	
R1	100	5773	0	0	481808	487580	431249	425477	0	Inverted	17	7	29.17%
R3	99.964	2795	1	0	363610	366404	123508	120714	0	Direct	64	54	45.76%
R77	100	7933	0	0	351656	359588	48220	40288	0	Direct	61	41	40.20%

**Figure 4:**
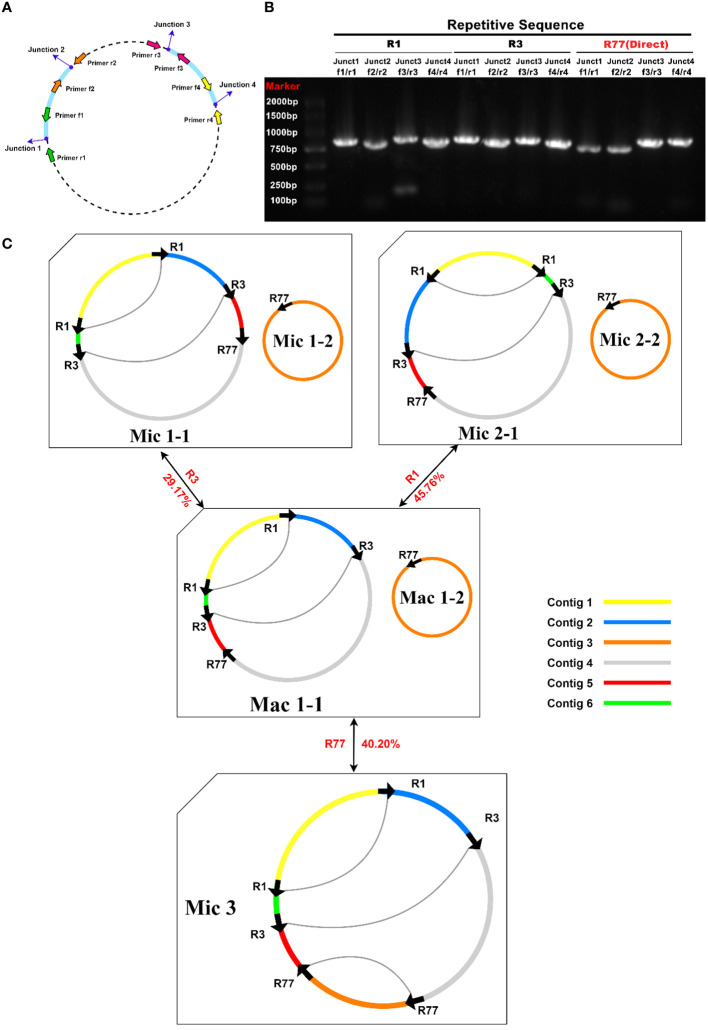
PCR validation of recombination products associated with repetitive sequence-mediated secondary configurations. **(A)** Schematic illustration of junctions related to each repetitive sequence. The corresponding primers are depicted as purple dots. F1-4: forward primers; R1-4: reverse primers. **(B)** Electrophoretic gel image of PCR products amplified using various forward and reverse primer combinations to amplify the DNA molecules corresponding to junctions 1-4. The name of the repetitive sequence, combinations of forward and reverse primers, expected junctions to be amplified, and lane numbers are displayed above the gel image. Each PCR product’s expected size encompasses those of the repetitive sequence and its 200-1000 bp long flanking sequences. The PCR product lengths are a rough evaluation of the successful amplification of fragments representing recombination products. **(C)** Hypothetical products of homologous recombination mediated by repetitive sequences R1, R3, and R77. Arrows indicate the repeat units of R1, R3, and R77. Arcs connect two repeat units if they are located on the same chromosome. Sequences surrounding the repeat units are displayed in distinct colors. Circles represent circular chromosomes. The genomic configuration is denoted by “C” followed by the configuration and chromosome numbers. Double-headed arrows indicate the source circular chromosomes, the repetitive elements, and the product circular chromosomes. The genomic configuration name is prefixed with “Ma,” representing “major” if it is the most abundant configuration; otherwise, the genomic configuration name is prefixed with “Mi,” representing “minor.” Mac is the genomic configuration containing chromosomes Mac1-1 and Mac1-2. Mac1-1 can undergo recombination mediated by R1 or R3 to form a circular chromosome Mic1-1 or Mic2-1. Mic3 only contains one circular chromosome, and it can undergo recombination mediated by R77 to form two circular chromosomes: Mac1-1 and Mac1-2.

**Figure 5:**
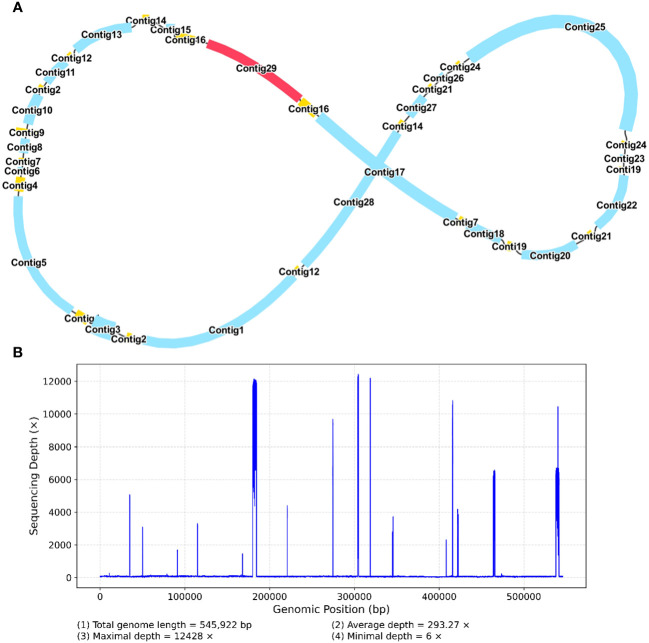
A schematic representation of the secondary configuration for the *R. glutinosa* mitogenome is provided. **(A)** A unitig graph for the *R. glutinosa* mitogenome was generated through *de novo* assembly of Illumina reads using Unicycler. This unitig graph consisted of seven contigs (depicted in yellow) that formed double bifurcating structures (DBSs) **(B)** The coverage depth of the Illumina short reads mapped to the *R. glutinosa* mitogenome sequences of the secondary configuration.

Primers were designed at each end of the repeat sequences further to investigate the recombination and potential configurations of R. glutinosa. Since the size of these repeat sequences exceeded 1,000 bp in length, specific primers were designed at the junction of each pair of repeats present on the primary single circular molecule (**Figure 4A**; **Supplementary Table S5**). In a recombinant configuration, PCR products (junctions 1-4) were shown in **Figure 4B**. The alignment of the Sanger sequencing results of the PCR products and the genomic sequences are shown in **Supplementary Figures S4**–**15**. We predicted the various configuration of the mitochondrial genome in **Figure 4C**. Three recombination events mediated by repeat sequences were confirmed (R1, R3, and R77, with R77 representing a pair of direct repeat sequences). All three sets of repeat sequences were found to generate secondary configurations, which is in accordance with according to the findings obtained through our long-read analysis (**Figure 5**; **Supplementary Figure S16**).

### 3.4 Mitochondrial plastid DNAs and mitochondrial nuclear DNAs analysis

**Figure 6:**
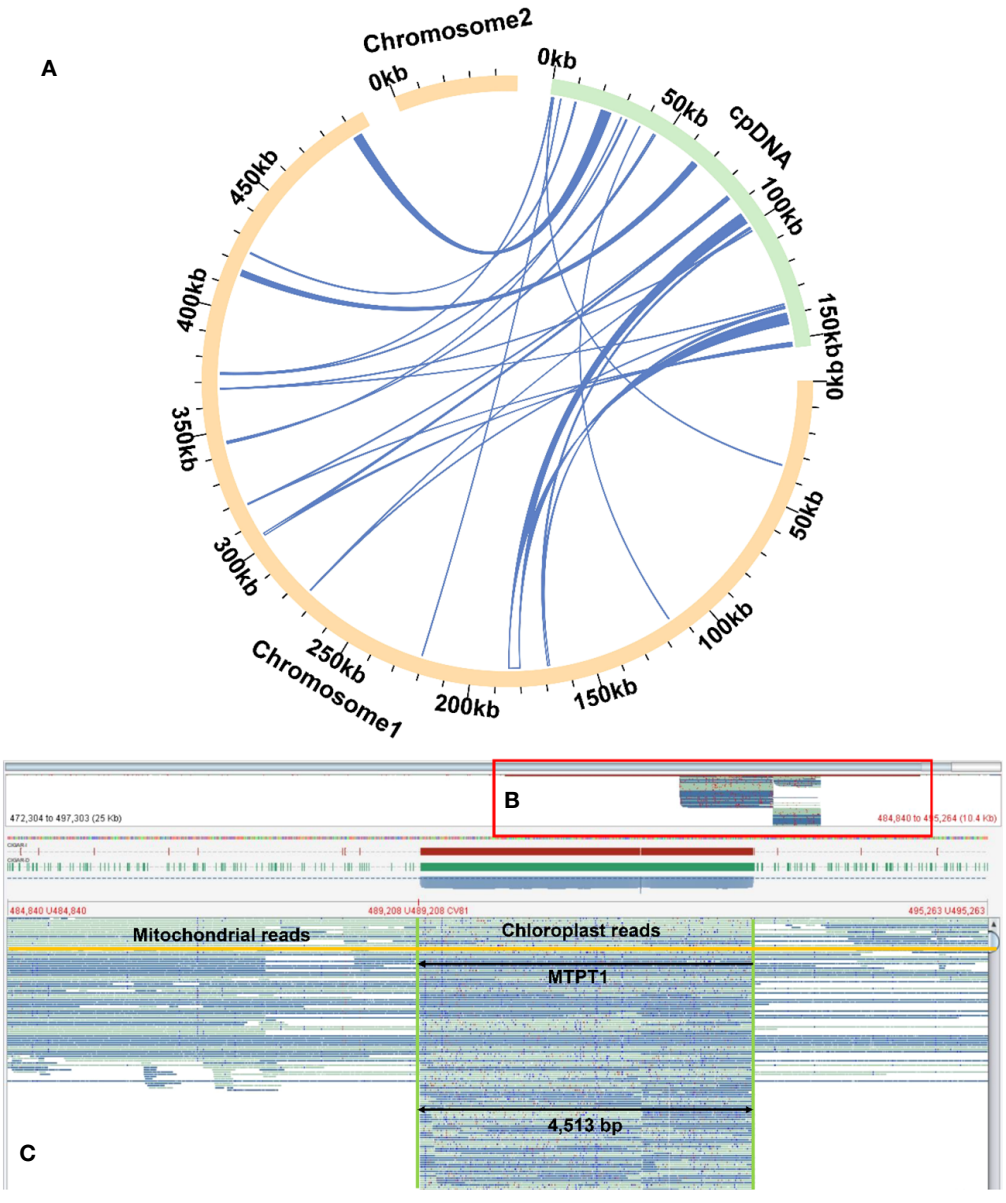
Examplar homologos sequences bewteen the mitogenome and chloroplastome. **(A)** Similar sequences are shared between the mitogenome and chloroplastome. The yellow and green arcs represent the mitogenome and chloroplastome genome (labeled as cpDNA), respectively. The inner circle arcs represent the MTPT fragments. **(B)** A bird’s eye view of MTPT, and the red box represents the enlarged part. **(C)** Mapping of long reads onto MTPT1 on chromosome 1. The MTPT sequence is highlighted in a green box. The encompassed regions illustrate upstream (mitoDNA) - MTPT - downstream (mitoDNA) sequences. A mitochondrial read is highlighted in yellow, bordered by mitoDNA sequences with MTPT sequence in the middle.

Homologous sequence transfer refers to the process in which a part of the chloroplast genome sequence that was integrated into the mitochondrial genome during the evolution (Brigulla and Wackernagel, 2010; Zhang G. J. et al., 2020). In the mitochondrial genome of *R. glutinosa*, we found 24 homologous DNA fragments, including six fragments from the chloroplast genome IR regions. The total length of these fragments was 13,685 bp, accounting for 2.51% of the whole mitochondrial genome, of which the longest fragment was 4,513 bp. We annotated these fragments and found that they contained a part of chloroplast genes (**Figure 6**; **Supplementary Figures S17**–**19**, **Supplementary Table S6**), including 12 complete genes (*ndhB, rps7, psbJ, psbL, psbF, psbE, rpl23, trnI-CAU, trnS-GGA, trnD-GUC, trnH-GUG* and *trnN-GUU*) and 11 partial genes (*trnL-CAA, rpoC1, rpoB, rpl2, trnI-GAU, psbA, psaB, trnK-UUU, rps4, ndhA* and *ndhB*).

Besides chloroplasts, there are homologous sequences between the mitochondria and nuclear genomes (**Supplementary Table S7**). Compared with the published whole genome sequence of *R. glutinosa*, we found that there were 4,395 fragments of nuclear DNAs with a total of 5,073,866bp length, which were similar. Among them, 3,694 fragments, with a total of 4,742,687 bp, were homologous to mitochondrial chromosome 1, with the longest fragment being 78,947 bp and the shortest being 36 bp. There are 1,701 fragments (331,179bp) homologous to mitochondrial chromosome 2 (the longest sequence had 32,990bp, and the shortest sequence was only 34bp). The total length of homologous fragments on these nuclear DNAs far exceeded the total length of the whole mitochondrial genome (545,523 bp), which might be related to the multiple migration of mitochondrial genes (Brigulla and Wackernagel, 2010; McFarlane and Humphrey, 2010; Knoll et al., 2014).

### 3.5 The phylogenomic analysis of *R. glutinosa*

**Figure 7:**
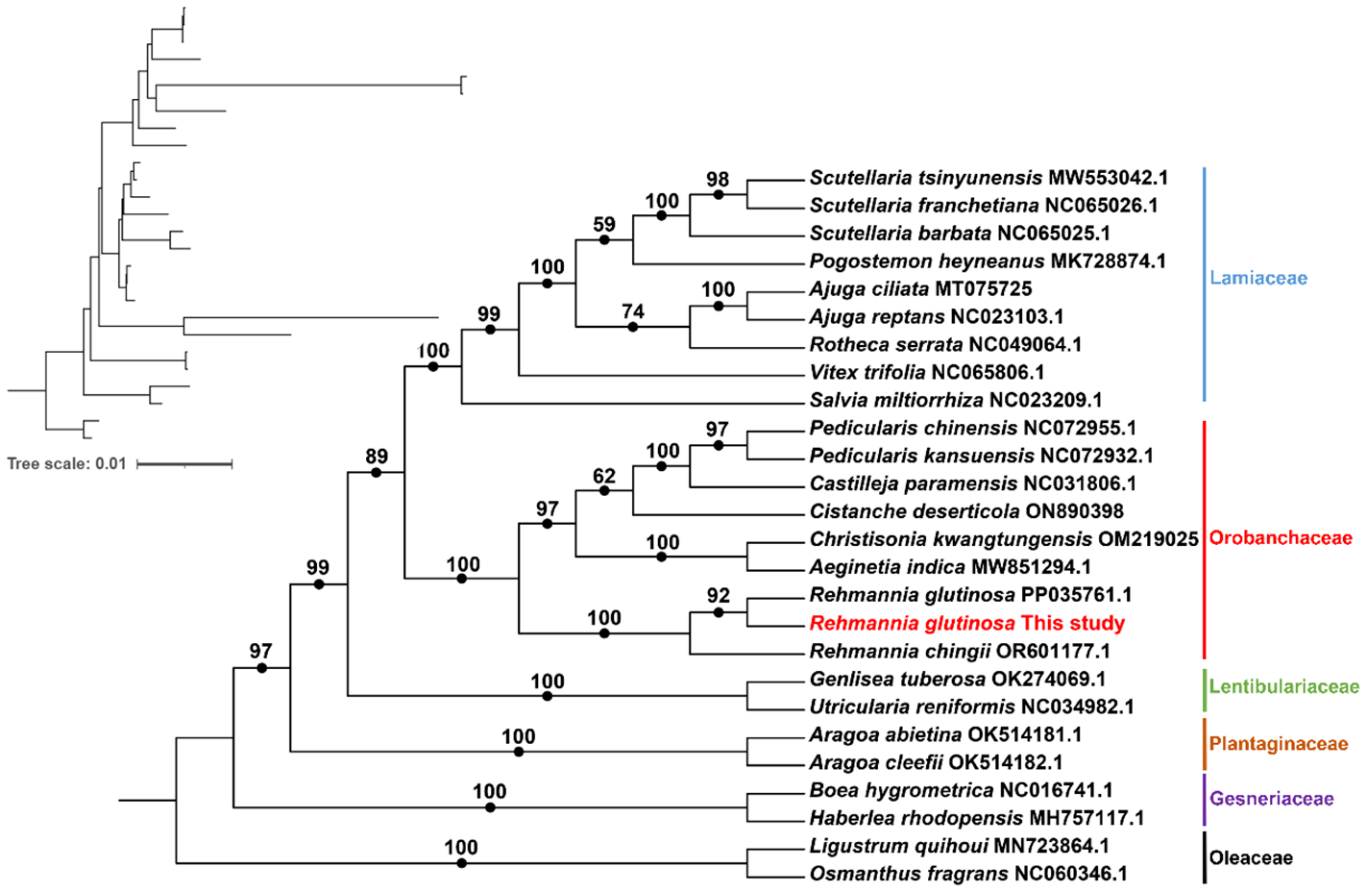
Molecular phylogenomic analysis of mitogenomes in Lamiales. The tree was constructed using concatenated conserved protein sequences from the mitogenomes of 21 species through maximum likelihood (ML) methods. Bootstrap scores were obtained using 1,000 replicates, and the ML bootstrap support values were indicated at the respective nodes. The tree in the upper left corner initially displays the original branch lengths. Two species from Oleaceae (*Ligustrum quihoui* and *Osmanthus fragrans*) were used as outgroups.

We conducted a phylogenetic analysis of the mitochondrial genomes of 21 Lamiales plants (*Rehmannia glutinosa* in our research: ON951335.1 – ON951336.1, *Rehmannia glutinosa* OM397952.2, *Rehmannia chingii* OR601177.1, *Castilleja paramensis* NC_031806.1, *Aeginetia indica* MW851294.1, *Christisonia kwangtungensis* OM219025_7.1, *Salvia miltiorrhiza* NC_023209.1, *Rotheca serrata* NC_049064.1, *Pogostemon heyneanus* MK728874.1, *Scutellaria tsinyunensis* MW553042.1, *Scutellaria barbata* NC_065025.1, *Scutellaria franchetiana* NC_065026.1, *Ajuga reptans* NC_023103.1, *Ajuga ciliata* MT075725_6.1, *Vitex trifolia* NC_065806.1, *Aragoa cleefii* OK514182.1, Aragoa abietina OK514181.1, *Boea hygrometrica* NC_016741.1, *Haberlea rhodopensis* MH757117.1, *Utricularia reniformis* NC_034982.1, *Genlisea tuberosa* voucher VFOM2001 OK274069.1, *Ligustrum quihoui* MN723864.1 and *Osmanthus fragrans* NC_060346.1). Two Oleaceae species (*Ligustrum quihoui* and *Osmanthus fragrans*) were selected as the outgroups. In total, the nucleotide sequences of 16 common genes (*atp1, atp4, ccmB, ccmC, ccmFC, ccmFN, cob, cox2, cox3, matR, nad1, nad2, nad3, nad5, nad6* and *rps13*) were used for phylogenetic analysis. As shown in **Figure 7**. The *R. glutinosa* mitogenome assembled by us and the mitogenome released on GenBank are grouped together, which can be identified as the same species.

### 3.6 The RNA editing sites in the mitogenome of *R. glutinosa*

**Figure 8:**
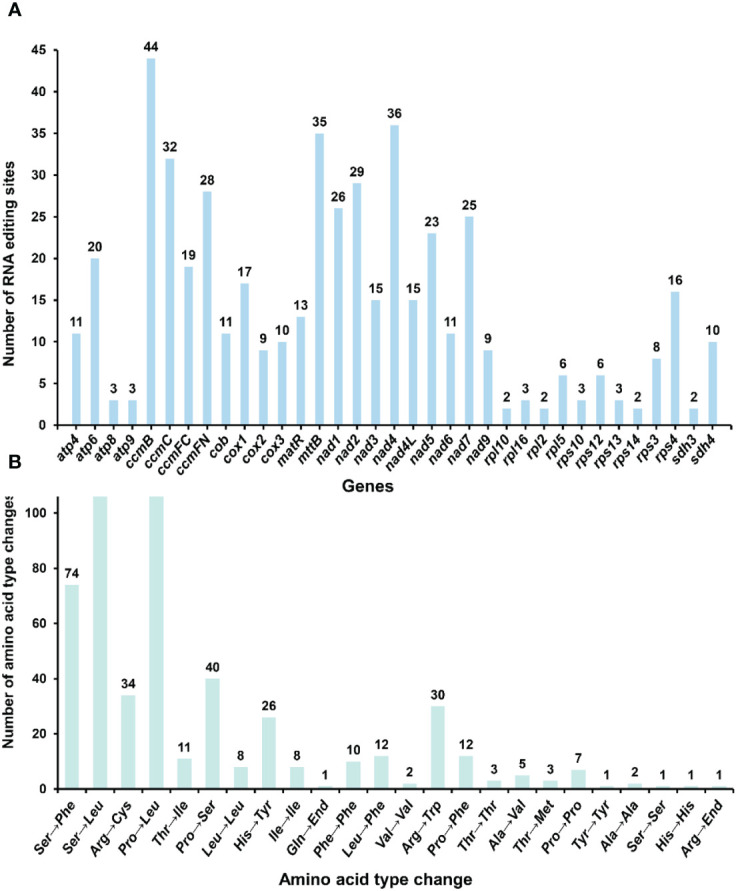
Statistics on the type and quantity of RNA editing events. **(A)** The number of RNA editing events for each gene. **(B)** The quantity of various amino acid changes.

RNA editing has been observed in the plant mitochondrial genomes extensively [18]. By mapping the transcriptome data to the reference mitogenome, we identified 507 RNA editing sites in the protein-coding regions (**Figure 8**; **Supplementary Table S8**). These genes include the genes *nad1, nad2, nad3, nad4, nad4L, nad5, nad6, nad7, nad9, cob, cox1, cox2, cox3, rpl2, rpl5, rpl10, rpl16, rps10, rps3, rps4, rps12, rps13, rps14, atp4, atp6, atp8, atp9, ccmB, ccmC, ccmFC, ccmFN, matR, mttB, sdh3* and *sdh4*. The types of all the editing sites were from C to U. Among them, the amino acid changes caused by non-synonymous substitution (15 of a total of 24 amino acid changes) may lead to the structural changes of the final synthesized protein. We detected RNA editing of two stop codons (*rps10* and *atp6*) and one start codon (*nad4L*) (**Supplementary Figures S20–22**), and the expression of related genes may be affected.

## 4 Discussion

### 4.1 Graph-based method for mitochondrial genome assembly

Early investigations into plant mitochondrial genomes postulated a single master circle configuration akin to chloroplast genomes (Wu et al., 2022). However, subsequent studies had revealed that a solitary reference genome is inadequate for representing the full extent of genetic variation between individuals, particularly in plant mitochondrial genomes (Backert et al., 1996; Gonzalez et al., 1999). Graph-based genomic representations have proven more effective in capturing configurational and structural variations, as demonstrated by the soybean pan-genome comprising 26 plant materials (Tian et al., 2012). Plant mitochondrial genomes exhibit substantial differences in complexity, size, and structure, and a single circular representation fails to encompass all potential configurations. Recent publications have presented graph-based plant mitochondrial genomes of Lamiales species, such as *Salvia miltiorrhiza* and *Scutellaria tsinyunensis* (Li et al., 2021; Yang H et al., 2022). These genomes comprise two chromosomal molecules, with nine configurations reported for *Salvia miltiorrhiza*. More graph-based assembly tools have been published, including Master graph and PMAT (He et al., 2023; Bi et al., 2024). Additionally, there is an increasing discovery of multi-conformation mitochondrial genomes. This study provides a graph-based mitochondrial genome consisting of 29 contigs, including ten repeat regions (DBS structure), from which additional minor configurations can emerge.

### 4.2 Multiple chromosome configurations and homologous recombination

Traditionally, plant mitochondrial genomes were considered single circular molecules, similar to chloroplast genomes, primarily because Illumina short-read sequencing technologies struggled to resolve complex bifurcated structures. Consequently, plant mitochondrial assemblies that failed to form a circle were often considered assembly errors (Sloan, 2013). However, Nanopore long-read sequencing and graph-based genome assembly approaches have provided new solutions for mitochondrial genome assembly, and the homologous recombination of mitochondrial genomes mediated by direct repeat sequences had been confirmed (Wang et al., 2024). These advancements have also facilitated better prediction the potential complex structures of the mitochondrial genomes.

The presence of minor configurations in mitochondrial genomes had been confirmed in various plant species, including *Scutellaria tsinyunensis, Ipomoea batatas, Saposhnikovia divaricata, Salvia miltiorrhiza, Cistanche deserticola*, and *Aeginetia indica* (Li et al., 2021; Miao et al., 2022; Ni et al., 2022; Yang H. et al., 2022; Yang Z. et al., 2022; Zhong et al., 2022). Among these, *Salvia miltiorrhiza* displayed two distinct mitochondrial configurations, which were ascribed to an increased occurrence of homologous recombination events (Yang H. et al., 2022). Our current investigation identified three minor configurations within the *R. glutinosa* mitochondrial genome, leveraging long-read sequencing technology. While we confirmed the junction sites through polymerase chain reaction (PCR) amplification and Sanger sequencing, dditional experimental evidence is necessary to validate this phenomenon further.

### 4.3 MTPT & NUMT

#### 4.3.1 NUMT

Mitochondria are thought to have originated from endosymbiotic α-proteobacteria, which subsequently experienced gene loss or transfer to the nucleus (Martin et al., 2015; Roger et al., 2017). The mitochondrial genome of flowering plants contains up to 40 known protein-coding genes, with the number of non-core genes varying significantly among species, apart from the 24 core protein-coding genes (Adams et al., 2002). One primary factor accounting for the variability in gene content within mitochondrial genomes is the transfer of mitochondrial genes to the nucleus during eukaryotic evolution (Brigulla and Wackernagel, 2010; McFarlane and Humphrey, 2010; Knoll et al., 2014). This functional gene transfer contributes to the co-evolution of mitochondria and the nucleus (Levin et al., 2014), has been found in mice and humans, an ongoing evolutionary process in land plants and some green algae. Due to the differing evolutionary and migration rates among plant species from various flora, gene migration frequencies also vary. For instance, the rps1 gene has been lost from the mitochondria of most Lamiales plants, a finding consistent with our study results.

For successful activation and expression of genes in the nucleus following physical transfer from mitochondria (Gualberto and Newton, 2017), these newly transferred genes must acquire promoters and other regulatory elements. If a protein lacks the necessary targeting information, it must obtain sequences of protein products targeting mitochondria. Several transferred genes have acquired mitochondrial target pre-sequences, which are removed from the protein following their introduction into mitochondria. Some genes have obtained mitochondrial pre-sequences from pre-existing mitochondrial protein genes (Liu et al., 2009; Gualberto and Newton, 2017). Once the transferred nuclear copy is activated, both this and the mitochondrial copies can be co-expressed for a period, at least at the transcript level, as demonstrated in the case of *cox*2 in some legumes, *rpl*5 in wheat, and *sdh*4 in poplar. The activation of transferred genes appears to be related to positive selection, but the existence of a nuclear screening mechanism for transferred genes remains uncertain. It is clear, however, that the transfer between mitochondria and the nucleus occurs frequently. In our study, we found that the nuclear DNA of *R. glutinosa* contained 4,329 fragments, with a total length of 4,880,380 bp, exhibiting similarity to mitochondrial sequences.

#### 4.3.2 MTPT

DNA transfer is prevalent in flowering plants, with DNA sequences being exchanged between the nuclear genome and the mitogenome (Wang X. C. et al., 2018). The most ancient mitochondrial-to-plastid DNA transfer (MTPT) events occurred approximately 300 million years ago, before the divergence of gymnosperms and angiosperms. Although most MTPTs are non-functional, some notable exceptions have been discovered, such as contributions to the replacement of tRNA genes, the creation of promoter regions and codons, and involvement in post-transcriptional RNA processing. Small DNA plastid fragments typically migrate to mitochondria, while larger fragments are exchanged between the nucleus and mitochondria.

In this study, the longest potential transfer fragment from the chloroplast of *R. glutinosa* was 4,513 bp, whereas the longest from mitochondria was 78,947 bp. The imperfect repair mechanism of mitochondria may facilitate the insertion of foreign sequences, and following the integration of nuclear organelle DNA, this DNA may undergo rearrangement, mutation, elimination, breakage, and proliferation. This process may represent one of the mechanisms driving species evolution.

### 4.4 RNA editing sites

RNA editing events typically occur during the post-transcriptional process in mitochondria, with specific RNA positions affected by RNA editing and their corresponding DNA positions referred to as editing sites (Edera et al., 2018). Early diverging lineages exhibit the highest number of editing sites among angiosperms, with approximately 400 editing sites reported in *Arabidopsis* (Edera et al., 2018). Our study verified 507 RNA editing events within the protein-coding region of *R. glutinosa*, all of which involved C-to-U conversions. The amino acid changes induced by this type of RNA editing may be statistically correlated with alterations in protein hydrophobicity. Cytoplasmic male sterility (CMS) is also associated with reduced, deleted, or incorrect RNA editing of mitochondrial gene transcripts, which modifies gene expression patterns and the functional properties of translation products, ultimately leading to CMS (Hu et al., 2014). For example, in male-sterile lines of Sorghum, the frequency of RNA editing within the *atp6* transcript is notably reduced. Additionally, two specific RNA editing sites within the *atp9* maintainer transcript in rice alter arginine codons to termination codons. Intriguingly, these amino acid changes result in alterations in the expression of three genes including two stop codons (*rps10* and *atp6*) and one start codon (*nad4L*). This finding provides insights for future molecular breeding of *R. glutinosa*.

Double-stranded DNA breaks (DSBs) are repaired primarily via two mechanisms: non-homologous end-joining (NHEJ) and homologous recombination (HR) (Roy et al., 2022). RNA can directly repair DSBs in an HR-dependent (RAD51-dependent) process, inhibited by RNases H1 and H2, known to degrade RNA-DNA hybrids (Mishra et al., 2018). Compared to other terrestrial plants, angiosperms have undergone extensive loss of editing sites through the substitution of editable cytidines with thymidines in their genomes. The homologous recombination of cDNA produced by reverse transcription of edited RNA appears to be one of the molecular mechanisms responsible for the loss of editing sites. While RNA editing is essential for DNA damage repair and genetic selection, its specific mechanism requires further investigation. The prediction and identification of these RNA editing sites offer valuable insights into inferring gene function through the introduction of novel codons. Furthermore, these findings highlight the crucial role of RNA editing in regulating mitochondrial gene expression in plants, particularly its impact on protein synthesis and functionality which subsequently influences plant growth and developmental processes.

## 5 Conclusion

In conclusion, this study offers a comprehensive analysis of the *R. glutinosa* mitochondrial genome, focusing on graph-based genome representation and identifying multiple chromosome configurations. Our findings reveal the presence of three minor configurations of the *R. glutinosa* mitochondrial genome, which were confirmed through PCR amplification and Sanger sequencing. Additionally, we observed the transfer of mitochondrial and chloroplast sequences to the nuclear genome, highlighting the complex interplay between organelle genomes and the nucleus. The research presented here contributes valuable insights into plant mitochondrial genomes’ intricate structure and dynamics, which can inform future molecular breeding efforts for *R. glutinosa* and other plant species. However, further experimental evidence is needed to fully understand the specific mechanisms of RNA editing and the potential nuclear screening processes for transferred genes. By expanding our understanding of plant mitochondrial genomes, we can better elucidate the factors that drive species evolution and develop targeted strategies for plant improvement.

## Data availability statement

The datasets presented in this study can be found in online repositories. The names of the repository/repositories and accession number(s) can be found below: GenBank (https://www.ncbi.nlm.nih.gov/) with accession numbers: OR030048.1 and ON951335.1 - ON951336.1, respectively. The associated BioProject, BioSample, and SRA numbers are PRJNA905540, SAMN37344158, and SRR26039035 for the Illumina sequencing reads and SRR26039034 for the Nanopore sequencing reads.

## Author contributions

TZ: Data curation, Formal analysis, Validation, Visualization, Writing – original draft. YN: Data curation, Methodology, Software, Writing – original draft. JL: Data curation, Methodology, Validation, Visualization, Writing – original draft. HC: Formal analysis, Writing – original draft. QL: Data curation, Validation, Writing – original draft. MJ: Data curation, Writing – original draft. LX: Funding acquisition, Supervision, Writing – review & editing. CL: Conceptualization, Funding acquisition, Supervision, Writing – review & editing. PX: Supervision, Writing – review & editing.

## References

[B1] Adams K. L. Qiu Y. L. Stoutemyer M. Palmer J. D. (2002). Punctuated evolution of mitochondrial gene content: High and variable rates of mitochondrial gene loss and transfer to the nucleus during angiosperm evolution. Proc. Natl. Acad. Sci. United States America 99, 9905–9912. doi: 10.1073/pnas.042694899 PMC126597 12119382

[B2] Backert S. Dorfel P. Lurz R. Borner T. (1996). Rolling-circle replication of mitochondrial DNA in the higher plant Chenopodium album (L). Mol. Cell. Biol. 16, 6285–6294. doi: 10.1128/MCB.16.11.6285 8887658 PMC231631

[B3] Bi C. Shen F. Han F. Qu Y. Hou J. Xu K.. (2024). PMAT: an efficient plant mitogenome assembly toolkit using low coverage HiFi sequencing data. Horticulture Res., 11(3), uhae023. doi: 10.1093/hr/uhae023 PMC10925850 38469379

[B4] Blomain E. S. McMahon S. B. (2012). Dynamic regulation of mitochondrial transcription as a mechanism of cellular adaptation. Biochim. Et Biophys. Acta-Gene Regul. Mech. 1819, 1075–1079. doi: 10.1016/j.bbagrm.2012.06.004 PMC6730671 22766037

[B5] Bolger A. M. Lohse M. Usadel B. (2014). Trimmomatic: a flexible trimmer for Illumina sequence data. Bioinf. (Oxford England) 30, 2114–2120. doi: 10.1093/bioinformatics/btu170 PMC4103590 24695404

[B6] Brigulla M. Wackernagel W. (2010). Molecular aspects of gene transfer and foreign DNA acquisition in prokaryotes with regard to safety issues. Appl. Microbiol. Biotechnol. 86, 1027–1041. doi: 10.1007/s00253-010-2489-3 20191269

[B7] Chateigner-Boutin A.-L. Small I. (2010). Plant RNA editing. RNA Biol. 7, 213–219. doi: 10.4161/rna.7.2.11343 20473038

[B8] Chen C. Chen H. Zhang Y. Thomas H. R. Frank M. H. He Y.. (2020). TBtools: an integrative toolkit developed for interactive analyses of big biological data. Mol. Plant 13, 1194–1202. doi: 10.1016/j.molp.2020.06.009 32585190

[B9] Chen J. Teng D. Wu Z. Li W. Feng Y. Tang Y.. (2021). Insights into the Molecular Mechanisms of Liuwei Dihuang Decoction via Network Pharmacology. Chem. Res. Toxicol. 34, 91–102. doi: 10.1021/acs.chemrestox.0c00359 33332098

[B10] Chen Y. Ye W. Zhang Y. Xu Y. (2015). High speed BLASTN: an accelerated MegaBLAST search tool. Nucleic Acids Res. 43, 7762–7768. doi: 10.1093/nar/gkv784 26250111 PMC4652774

[B11] Chevigny N. Schatz-Daas D. Lotfi F. Gualberto J. M. (2020). DNA repair and the stability of the plant mitochondrial genome. Int. J. Mol. Sci. 21. doi: 10.3390/ijms21010328 PMC6981420 31947741

[B12] Dahal S. Dubey S. Raghavan S. C. (2018). Homologous recombination-mediated repair of DNA double-strand breaks operates in mammalian mitochondria. Cell. Mol. Life Sci. 75, 1641–1655. doi: 10.1007/s00018-017-2702-y 29116362 PMC11105789

[B13] Edera A. A. Gandini C. L. Virginia Sanchez-Puerta M. (2018). Towards a comprehensive picture of C-to-U RNA editing sites in angiosperm mitochondria. Plant Mol. Biol. 97, 215–231. doi: 10.1007/s11103-018-0734-9 29761268

[B14] Fernie A. R. Yang J. (2019). *De novo* domestication: an alternative route toward new crops for the future. Mol. Plant 12, 615–631. doi: 10.1016/j.molp.2019.03.016 30999078

[B15] Gonzalez P. Barroso G. Labarere J. (1999). Molecular gene organisation and secondary structure of the mitochondrial large subunit ribosomal RNA from the cultivated Basidiomycota Agrocybe aegerita: a 13 kb gene possessing six unusual nucleotide extensions and eight introns. Nucleic Acids Res. 27, 1754–1761. doi: 10.1093/nar/27.7.1754 10076008 PMC148380

[B16] Greiner S. Lehwark P. Bock R. (2019). OrganellarGenomeDRAW (OGDRAW) version 1.3.1: expanded toolkit for the graphical visualization of organellar genomes. Nucleic Acids Res. 47, W59–W64. 30949694 10.1093/nar/gkz238 PMC6602502

[B17] Gualberto J. M. Newton K. J. (2017). Plant mitochondrial genomes: dynamics and mechanisms of mutation. Annu. Rev. Plant Biol. 68, 225–252. doi: 10.1146/annurev-arplant-043015-112232 28226235

[B18] He W. Xiang K. Chen C. Wang J. Wu Z. (2023). Master graph: an essential integrated assembly model for the plant mitogenome based on a graph-based framework. Briefings Bioinf. 24, bbac522. doi: 10.1093/bib/bbac522 36644898

[B19] Hu J. Huang W. Huang Q. Qin X. Yu C. Wang L.. (2014). Mitochondria and cytoplasmic male sterility in plants. Mitochondrion 19, 282–288. doi: 10.1016/j.mito.2014.02.008 24566371

[B20] Ichinose M. Sugita M. (2017). RNA editing and its molecular mechanism in plant organelles. Genes 8. 10.3390/genes8010005 PMC5295000 28025543

[B21] Igarashi K. Kazama T. Motomura K. Toriyama K. (2013). Whole genomic sequencing of RT98 mitochondria derived from oryza rufipogon and northern blot analysis to uncover a cytoplasmic male sterility-associated gene. Plant Cell Physiol. 54, 237–243. doi: 10.1093/pcp/pcs177 23248202

[B22] Jin J.-J. Yu W.-B. Yang J.-B. Song Y. dePamphilis C. W. Yi T.-S.. (2020). GetOrganelle: a fast and versatile toolkit for accurate *de novo* assembly of organelle genomes. Genome Biol. 21. doi: 10.1186/s13059-020-02154-5 PMC7488116 32912315

[B23] Kang B.-C. Bae S.-J. Lee S. Lee J. S. Kim A. Lee H.. (2021). Chloroplast and mitochondrial DNA editing in plants. Nat. Plants 7, 899–905. doi: 10.1038/s41477-021-00943-9 34211132 PMC8289734

[B24] Kim Y.-G. Komakech R. Jeong D. H. Park Y. M. Lee T. K. Kim K. H.. (2020). Verification of the field productivity of rehmannia glutinosa (Gaertn.) DC. Developed through optimized *in vitro* culture method. Plants-Basel 9. 10.3390/plants9030317 PMC7154825 32138268

[B25] Knoll A. Fauser F. Puchta H. (2014). DNA recombination in somatic plant cells: mechanisms and evolutionary consequences. Chromosome Res. 22, 191–201. doi: 10.1007/s10577-014-9415-y 24788060

[B26] Koenig D. Jimenez-Gomez J. M. Kimura S. Fulop D. Chitwood D. H. Headland L. R.. (2013). Comparative transcriptomics reveals patterns of selection in domesticated and wild tomato. Proc. Natl. Acad. Sci. United States America 110, E2655–E2662. doi: 10.1073/pnas.1309606110 PMC3710864 23803858

[B27] Letunic I. Bork P. (2021). Interactive Tree Of Life (iTOL) v5: an online tool for phylogenetic tree display and annotation. Nucleic Acids Res. 49, W293–W296. doi: 10.1093/nar/gkab301 33885785 PMC8265157

[B28] Levin L. Blumberg A. Barshad G. Mishmar D. (2014). Mito-nuclear co-evolution: the positive and negative sides of functional ancient mutations. Front. Genet. 5, 448. doi: 10.3389/fgene.2014.00448 25566330 PMC4274989

[B29] Li H. Durbin R. (2010). Fast and accurate long-read alignment with Burrows-Wheeler transform. Bioinf. (Oxford England) 26, 589–595. doi: 10.1093/bioinformatics/btp698 PMC2828108 20080505

[B30] Li H. Handsaker B. Wysoker A. Fennell T. Ruan J. Homer N.. (2009). The sequence alignment/map format and SAMtools. Bioinf. (Oxford England) 25, 2078–2079. doi: 10.1093/bioinformatics/btp352 PMC2723002 19505943

[B31] Li J. Xu Y. Shan Y. Pei X. Yong S. Liu C.. (2021). Assembly of the complete mitochondrial genome of an endemic plant, Scutellaria tsinyunensis, revealed the existence of two conformations generated by a repeat-mediated recombination. Planta 254. doi: 10.1007/s00425-021-03684-3 34302538

[B32] Li M. Jiang H. Hao Y. Du K. Du H. Ma C.. (2022). A systematic review on botany, processing, application, phytochemistry and pharmacological action of Radix Rehmnniae. J. Ethnopharmacol 285. doi: 10.1016/j.jep.2021.114820 34767834

[B33] Liao S. Chen L. Song Z. He H. (2022). The fate of damaged mitochondrial DNA in the cell. Biochim. Et Biophys. Acta-Molecular Cell Res. 1869. doi: 10.1016/j.bbamcr.2022.119233 35131372

[B34] Liu C. Ma R. Wang L. Zhu R. Liu H. Guo Y.. (2017). Rehmanniae Radix in osteoporosis: A review of traditional Chinese medicinal uses, phytochemistry, pharmacokinetics and pharmacology. J. Ethnopharmacol 198, 351–362. doi: 10.1016/j.jep.2017.01.021 28111216

[B35] Liu S. Ni Y. Li J. Zhang X. Yang H. Chen H.. (2023). CPGView: A package for visualizing detailed chloroplast genome structures. Mol. Ecol. Resour. doi: 10.1111/1755-0998.13729 36587992

[B36] Liu S.-L. Zhuang Y. Zhang P. Adams K. L. (2009). Comparative analysis of structural diversity and sequence evolution in plant mitochondrial genes transferred to the nucleus. Mol. Biol. Evol. 26, 875–891. doi: 10.1093/molbev/msp011 19168566

[B37] Lowe T. M. Eddy S. R. (1997). tRNAscan-SE: A program for improved detection of transfer RNA genes in genomic sequence. Nucleic Acids Res. 25, 955–964. doi: 10.1093/nar/25.5.955 9023104 PMC146525

[B38] Lu Z. Huang M. Lin H. Wang G. Li H. (2022). Network pharmacology and molecular docking approach to elucidate the mechanisms of Liuwei Dihuang pill in diabetic osteoporosis. J. Orthopaedic Surg. Res. 17. doi: 10.1186/s13018-022-03194-2 PMC9195436 35701780

[B39] Martin W. F. Garg S. Zimorski V. (2015). Endosymbiotic theories for eukaryote origin. Philos. Trans. R. Soc. B-Biological Sci. 370. doi: 10.1098/rstb.2014.0330 PMC4571569 26323761

[B40] McFarlane R. J. Humphrey T. C. (2010). A role for recombination in centromere function. Trends Genet. 26, 209–213. doi: 10.1016/j.tig.2010.02.005 20382440

[B41] Meng X. He M. Guo R. Duan R. Huo F. Lv C.. (2017). Investigation of the effect of the degree of processing of radix rehmanniae preparata (Shu dihuang) on shu dihuangtan carbonization preparation technology. Molecules 22. doi: 10.3390/molecules22071193 PMC6152270 28718784

[B42] Meyer R. S. Purugganan M. D. (2013). Evolution of crop species: genetics of domestication and diversification. Nat. Rev. Genet. 14, 840–852. doi: 10.1038/nrg3605 24240513

[B43] Miao Y. Chen H. Xu W. Liu C. Huang L. (2022). Cistanche species mitogenomes suggest diversity and complexity in lamiales-order mitogenomes. Genes 13. doi: 10.3390/genes13101791 PMC9602076 36292676

[B44] Milne I. Bayer M. Cardle L. Shaw P. Stephen G. Wright F.. (2010). Tablet-next generation sequence assembly visualization. Bioinf. (Oxford England) 26, 401–402. doi: 10.1093/bioinformatics/btp666 PMC2815658 19965881

[B45] Mishra A. Saxena S. Kaushal A. Nagaraju G. (2018). RAD51C/XRCC3 facilitates mitochondrial DNA replication and maintains integrity of the mitochondrial genome. Mol. Cell. Biol. 38. doi: 10.1128/MCB.00489-17 PMC5770535 29158291

[B46] Misra S. Harris N. (2006). Using Apollo to browse and edit genome annotations. Curr. Protoc. Bioinf. 10.1002/0471250953.bi0905s12 18428771

[B47] Mohammed T. Firoz A. Ramadan A. M. M. (2022). RNA editing in chloroplast: advancements and opportunities. Curr. Issues Mol. Biol. 44, 5593–5604. doi: 10.3390/cimb44110379 36421663 PMC9688838

[B48] Ni Y. Li J. Chen H. Yue J. Chen P. Liu C. (2022). Comparative analysis of the chloroplast and mitochondrial genomes of Saposhnikovia divaricata revealed the possible transfer of plastome repeat regions into the mitogenome. BMC Genomics 23. doi: 10.1186/s12864-022-08821-0 PMC9364500 35945507

[B49] Okazaki M. Kazama T. Murata H. Motomura K. Toriyama K. (2013). Whole mitochondrial genome sequencing and transcriptional analysis to uncover an RT102-type cytoplasmic male sterility-associated candidate gene derived from oryza rufipogon. Plant Cell Physiol. 54, 1560–1568. doi: 10.1093/pcp/pct102 23852329

[B50] Osellame L. D. Blacker T. S. Duchen M. R. (2012). Cellular and molecular mechanisms of mitochondrial function. Best Pract. Res. Clin. Endocrinol. Metab. 26, 711–723. 23168274 10.1016/j.beem.2012.05.003 PMC3513836

[B51] Palozzi J. M. Jeedigunta S. P. Minenkova A. Monteiro V. L. Thompson Z. S. Lieber T.. (2022). Mitochondrial DNA quality control in the female germline requires a unique programmed mitophagy. Cell Metab. 34, 1809. doi: 10.1016/j.cmet.2022.10.005 36323236

[B52] Picardi E. Pesole G. (2013). REDItools: high-throughput RNA editing detection made easy. Bioinf. (Oxford England) 29, 1813–1814. doi: 10.1093/bioinformatics/btt287 23742983

[B53] Roger A. J. Munoz-Gomez S. A. Kamikawa R. (2017). The origin and diversification of mitochondria. Curr. Biol. 27, R1177–R1192. doi: 10.1016/j.cub.2017.09.015 29112874

[B54] Roy A. Kandettu A. Ray S. Chakrabarty S. (2022). Mitochondrial DNA replication and repair defects: Clinical phenotypes and therapeutic interventions. Biochim. Et Biophys. Acta-Bioenergetics 1863. doi: 10.1016/j.bbabio.2022.148554 35341749

[B55] Rozewicki J. Li S. Amada K. M. Standley D. M. Katoh K. (2019). MAFFT-DASH: integrated protein sequence and structural alignment. Nucleic Acids Res. 47, W5–W10. doi: 10.1093/nar/gkz342 31062021 PMC6602451

[B56] Shi L. Chen H. Jiang M. Wang L. Wu X. Huang L.. (2019). CPGAVAS2, an integrated plastome sequence annotator and analyzer. Nucleic Acids Res. 47, W65–W73. doi: 10.1093/nar/gkz345 31066451 PMC6602467

[B57] Sloan D. B. (2013). One ring to rule them all? Genome sequencing provides new insights into the ‘master circle’model of plant mitochondrial DNA structure. New Phytol. 200, 978–985. doi: 10.1111/nph.12395 24712049

[B58] Small I. D. Schallenberg-Ruedinger M. Takenaka M. Mireau H. Ostersetzer-Biran O. (2020). Plant organellar RNA editing: what 30 years of research has revealed. Plant J. 101, 1040–1056. doi: 10.1111/tpj.14578 31630458

[B59] Sun T. Bentolila S. Hanson M. R. (2016). The unexpected diversity of plant organelle RNA editosomes. Trends Plant Sci. 21, 962–973. doi: 10.1016/j.tplants.2016.07.005 27491516

[B60] Takenaka M. Verbitskly D. van der Merwe J. A. Zehrmann A. Brennicke A. (2008). The process of RNA editing in plant mitochondria. Mitochondrion 8, 35–46. doi: 10.1016/j.mito.2007.09.004 18326075

[B61] Tian C. F. Zhou Y. J. Zhang Y. M. Li Q. Q. Zhang Y. Z. Li D. F.. (2012). Comparative genomics of rhizobia nodulating soybean suggests extensive recruitment of lineage-specific genes in adaptations. Proc. Natl. Acad. Sci. United States America 109, 8629–8634. 10.1073/pnas.1120436109 PMC3365164 22586130

[B62] Tillich M. Lehwark P. Pellizzer T. Ulbricht-Jones E. S. Fischer A. Bock R.. (2017). GeSeq - versatile and accurate annotation of organelle genomes. Nucleic Acids Res. 45, W6–W11. doi: 10.1093/nar/gkx391 28486635 PMC5570176

[B63] Untergasser A. Cutcutache I. Koressaar T. Ye J. Faircloth B. C. Remm M.. (2012). Primer3-new capabilities and interfaces. Nucleic Acids Res. 40. doi: 10.1093/nar/gks596 PMC3424584 22730293

[B64] Wang X.-C. Chen H. Yang D. Liu C. (2018). Diversity of mitochondrial plastid DNAs (MTPTs) in seed plants. Mitochondrial DNA Part A 29, 635–642. doi: 10.1080/24701394.2017.1334772 28573928

[B65] Wang R. Wang Y. Yang Q. Kang C. Li M. (2018). Unraveling the characteristics of the microbial community and potential pathogens in the rhizosphere soil of Rehmannia glutinosa with root rot disease. Appl. Soil Ecol. 130, 271–279. doi: 10.1016/j.apsoil.2018.07.001

[B66] Wang J. Kan S. Liao X. Zhou J. Tembrock L. R. Daniell H.. (2024). Plant organellar genomes: Much done, much more to do. Trends Plant Sci. doi: 10.1016/j.tplants.2023.12.014 38220520

[B67] Wick R. Judd L. M. Gorrie C. L. Holt K. E. (2017). Completing bacterial genome assemblies with multiplex MinION sequencing. Microb. Genom. 3 (10), e000132. doi: 10.1099/mgen.0.000132 29177090 PMC5695209

[B68] Wick R. R. Judd L. M. Gorrie C. L. Holt K. E. (2017). Unicycler: Resolving bacterial genome assemblies from short and long sequencing reads. PloS Comput. Biol. 13. doi: 10.1371/journal.pcbi.1005595 PMC5481147 28594827

[B69] Wu B. Chen H. M. Shao J. J. Zhang H. Wu K. Liu C. (2017). Identification of symmetrical RNA editing events in the mitochondria of salvia miltiorrhiza by strand-specific RNA sequencing. Sci. Rep. 7, 11. doi: 10.1038/s41598-017-00052-8 28186130 PMC5301482

[B70] Wu P. Chen H. Xu C. Yang J. Zhang X. C. Zhou S. L. (2021). NOVOWrap: An automated solution for plastid genome assembly and structure standardization. Mol. Ecol. Resour. 21, 2177–2186. doi: 10.1111/1755-0998.13410 33934526

[B71] Wu Z.-Q. Liao X.-Z. Zhang X.-N. Tembrock L. R. Broz A. (2022). Genomic architectural variation of plant mitochondria-A review of multichromosomal structuring. J. Syst. Evol. 60, 160–168. doi: 10.1111/jse.12655

[B72] Yang H. Chen H. Ni Y. Li J. Cai Y. Ma B.. (2022). *De novo* hybrid assembly of the salvia miltiorrhiza mitochondrial genome provides the first evidence of the multi-chromosomal mitochondrial DNA structure of salvia species. Int. J. Mol. Sci. 23. doi: 10.3390/ijms232214267 PMC9694629 36430747

[B73] Yang Z. Ni Y. Lin Z. Yang L. Chen G. Nijiati N.. (2022). *De novo* assembly of the complete mitochondrial genome of sweet potato (Ipomoea batatas L. Lam) revealed the existence of homologous conformations generated by the repeat-mediated recombination. BMC Plant Biol. 22. 10.1186/s12870-022-03665-y PMC9185937 35681138

[B74] Zhang G.-J. Dong R. Lan L.-N. Li S.-F. Gao W.-J. Niu H.-X. (2020). Nuclear integrants of organellar DNA contribute to genome structure and evolution in plants. Int. J. Mol. Sci. 21. doi: 10.3390/ijms21030707 PMC7037861 31973163

[B75] Zhang D. Gao F. Jakovlic I. Zou H. Zhang J. Li W. X.. (2020). PhyloSuite: An integrated and scalable desktop platform for streamlined molecular sequence data management and evolutionary phylogenetics studies. Mol. Ecol. Resour. 20, 348–355. doi: 10.1111/1755-0998.13096 31599058

[B76] Zheng W. Wang G. Zhang Z. Wang Z. Ma K. (2020). Research progress on classical traditional Chinese medicine formula Liuwei Dihuang pills in the treatment of type 2 diabetes. Biomedicine Pharmacotherapy 121. doi: 10.1016/j.biopha.2019.109564 31683180

[B77] Zhong Y. Yu R. Chen J. Liu Y. Zhou R. (2022). Highly active repeat-mediated recombination in the mitogenome of the holoparasitic plant Aeginetia indica. Front. Plant Sci. 13. doi: 10.3389/fpls.2022.988368 PMC9532969 36212306

