# Supplementary material for: Comprehensive analysis of the mitochondrial genome of Rehmannia glutinosa: insights into repeat-mediated recombinations and RNA editing-induced stop codon acquisition
Source: Front Plant Sci. 2024 May 14;15:1326387. doi: 10.3389/fpls.2024.1326387 (PMC11130359; doi:10.3389/fpls.2024.1326387)

**Figure S1**. The coverage depth of the Illumina short reads mapped to the *R. glutinosa* mitogenome sequences. (A) Mitogenome chormosome 1 (B) Mitogenome chormosome 2

**
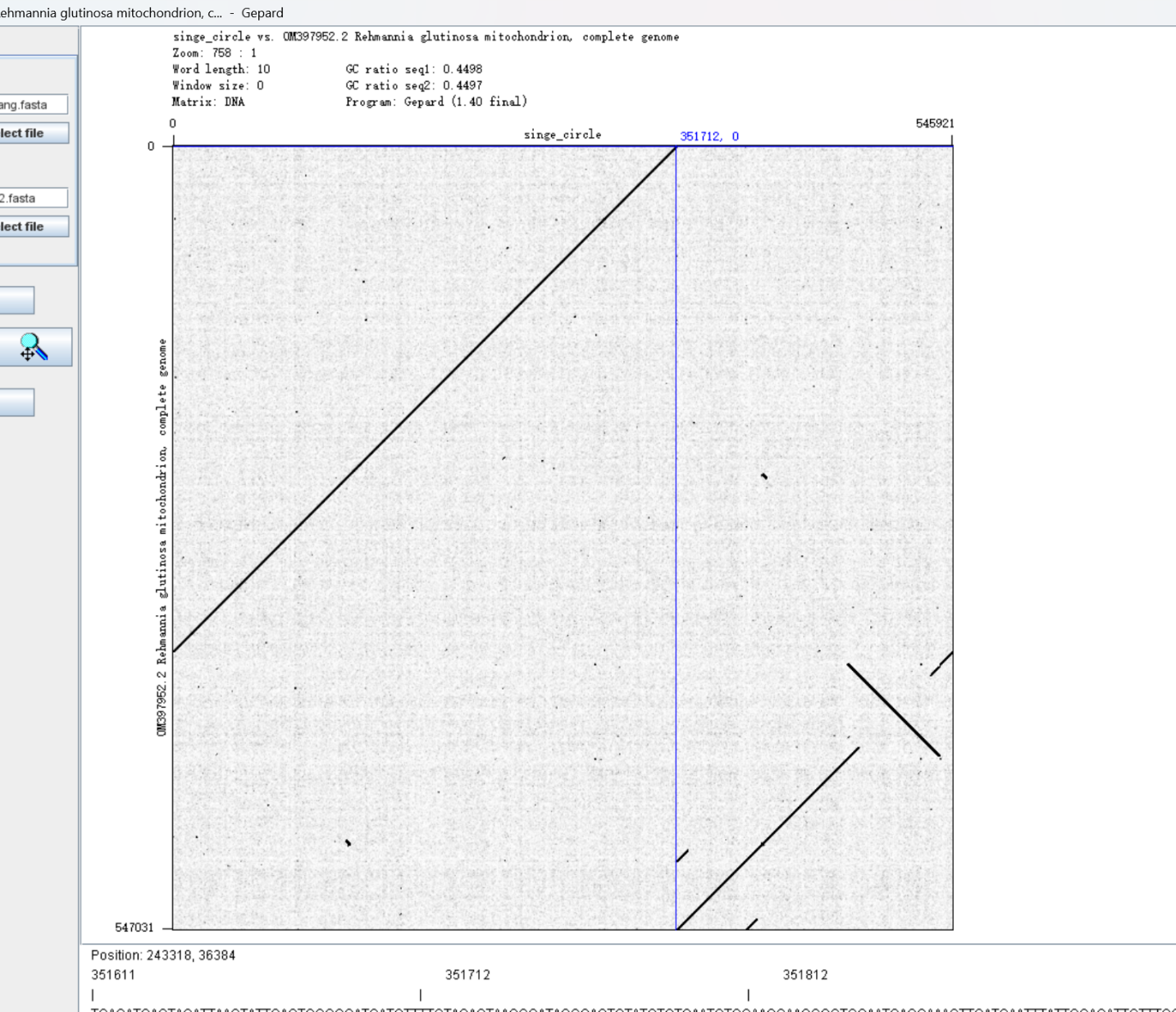
**

**Figure S2**. The coverage depth of the Illumina short reads mapped to the *R. glutinosa* mitogenome sequences. (A) Mitogenome chormosome 1 (B) Mitogenome chormosome 2

**
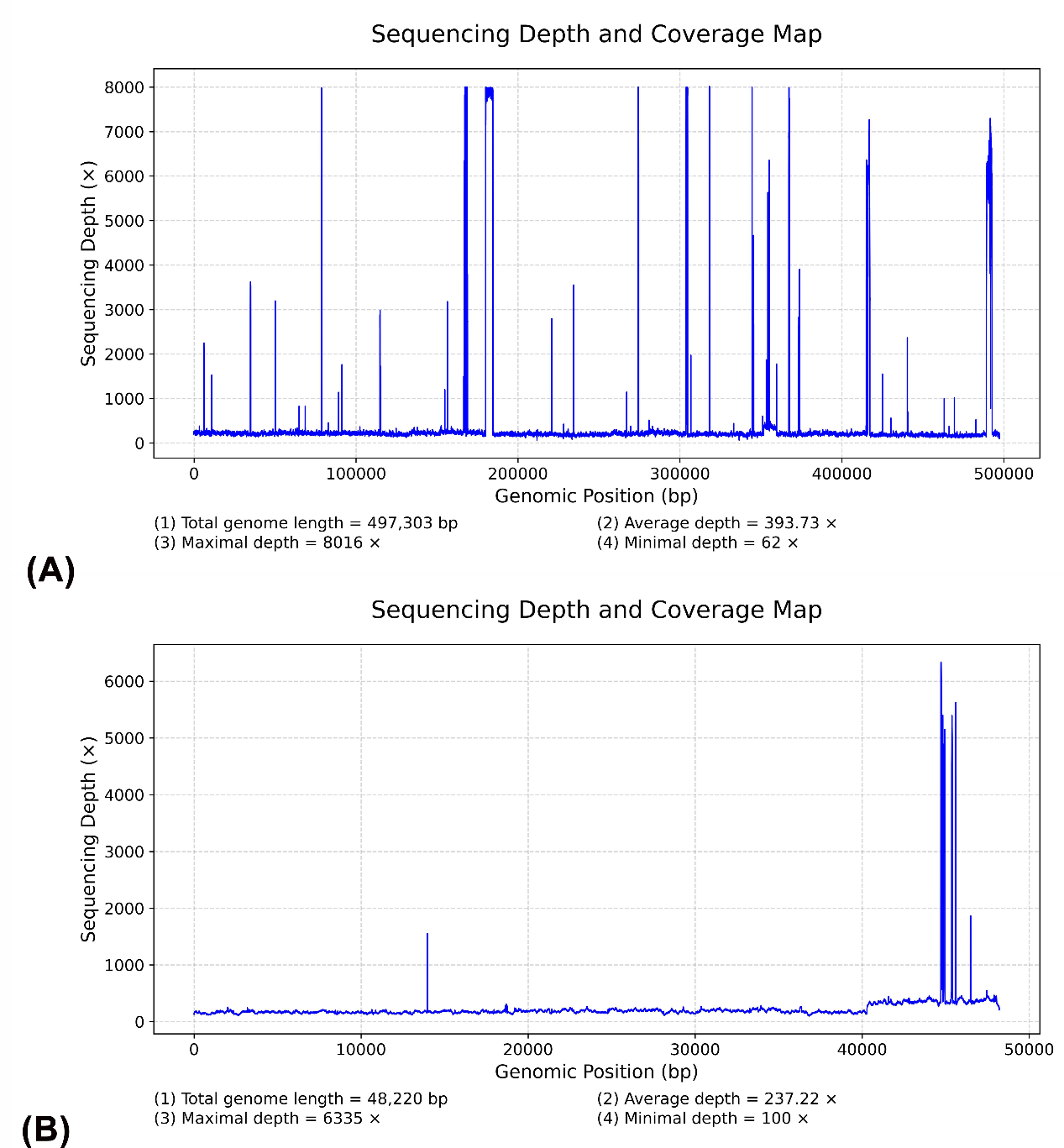
**

**Figure S3**. The coverage depth of the Nanopore long reads mapped to the *R. glutinosa* mitogenome sequences. (A) Mitogenome chormosome 1 (B) Mitogenome chormosome 2

**
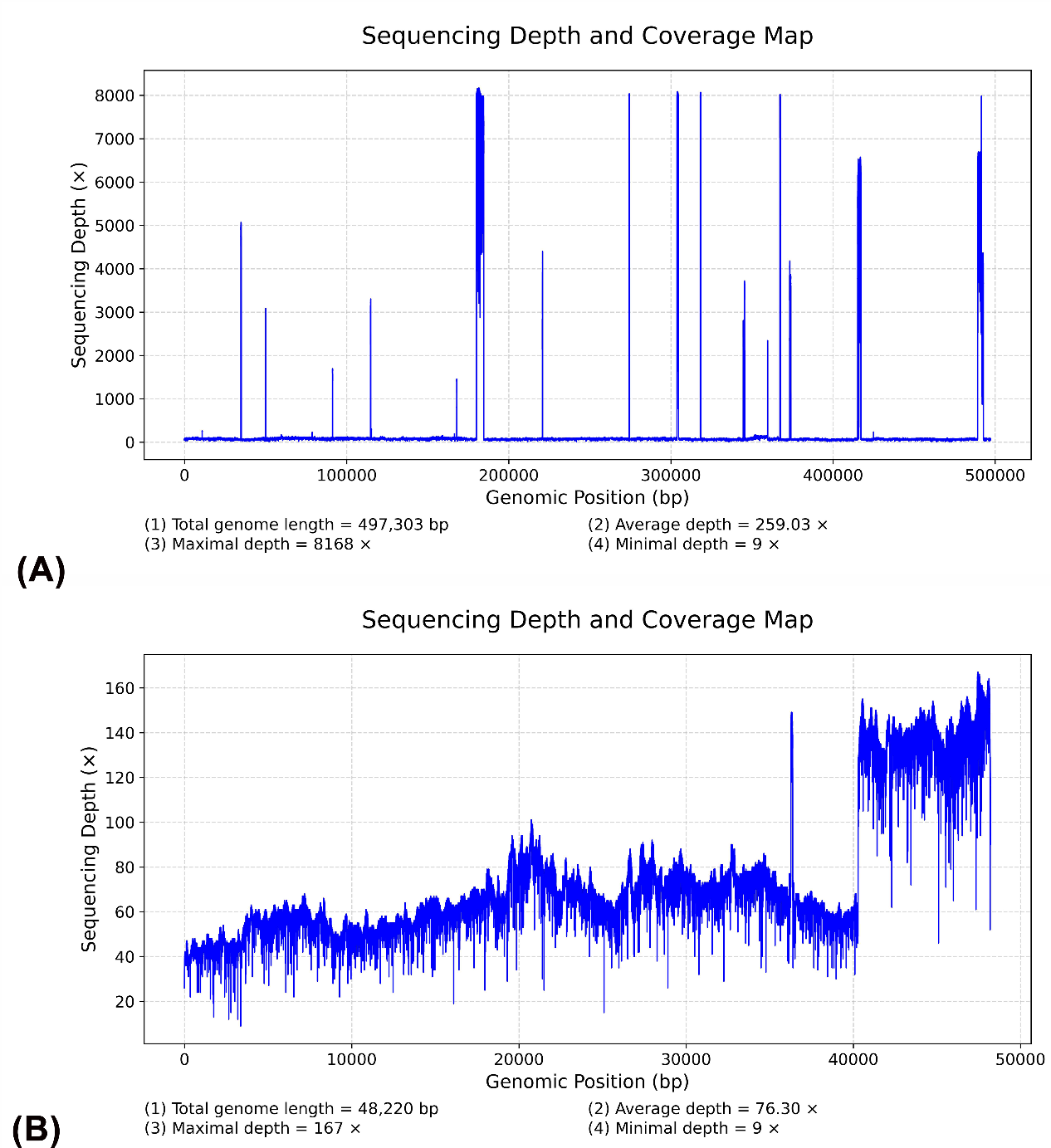
**

**Figure S4**. The results of Sanger sequencing for Junction 1 (f1/r1) of repeat sequence 1 (R1). The sequence at the top, in the middle and at the bottom are the assembled genome sequence, the sequence of the PCR products, and the consensus sequence.


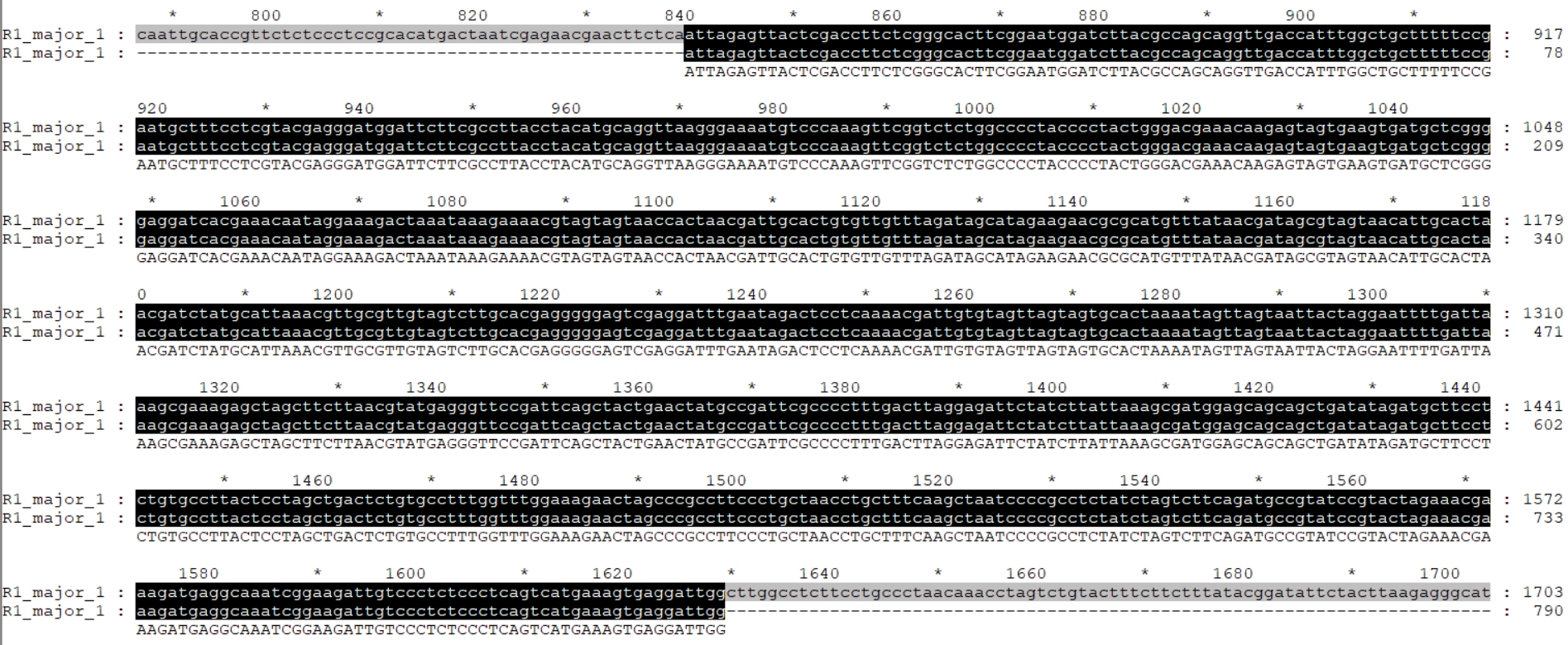


**Figure S5**. The results of Sanger sequencing for Junction 2 (f2/r2) of repeat sequence 1 (R1). The sequence at the top, in the middle and at the bottom are the assembled genome sequence, the sequence of the PCR products, and the consensus sequence.


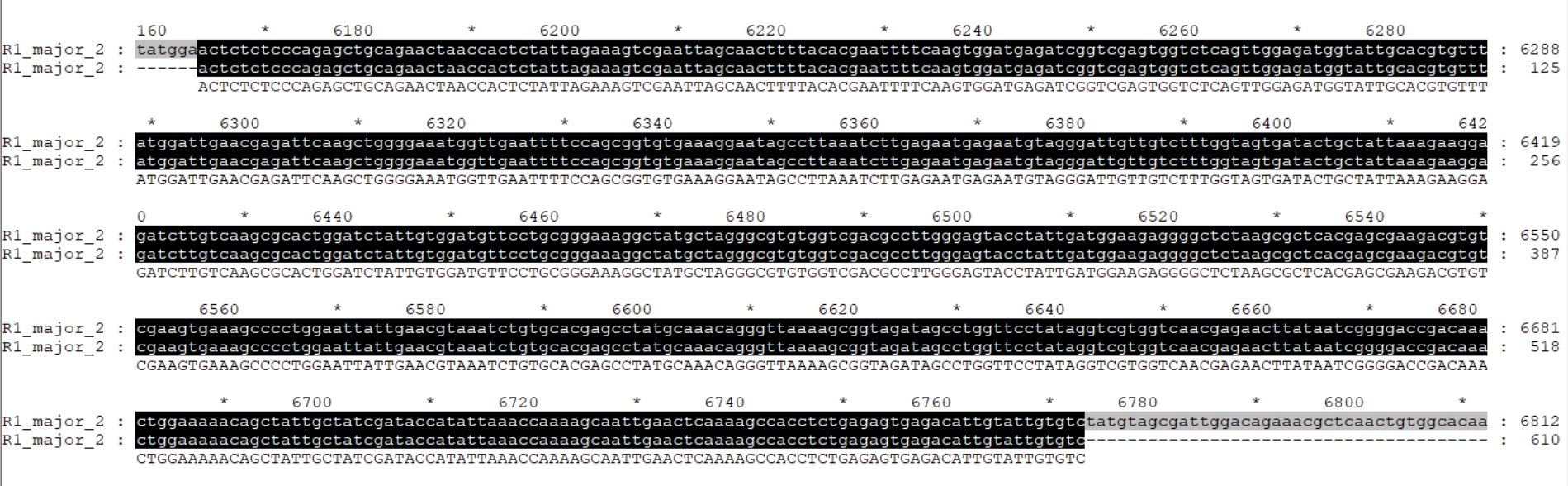


**Figure S6**. The results of Sanger sequencing for Junction 3 (f3/r3) of repeat sequence 1 (R1). The sequence at the top, in the middle and at the bottom are the assembled genome sequence, the sequence of the PCR products, and the consensus sequence.


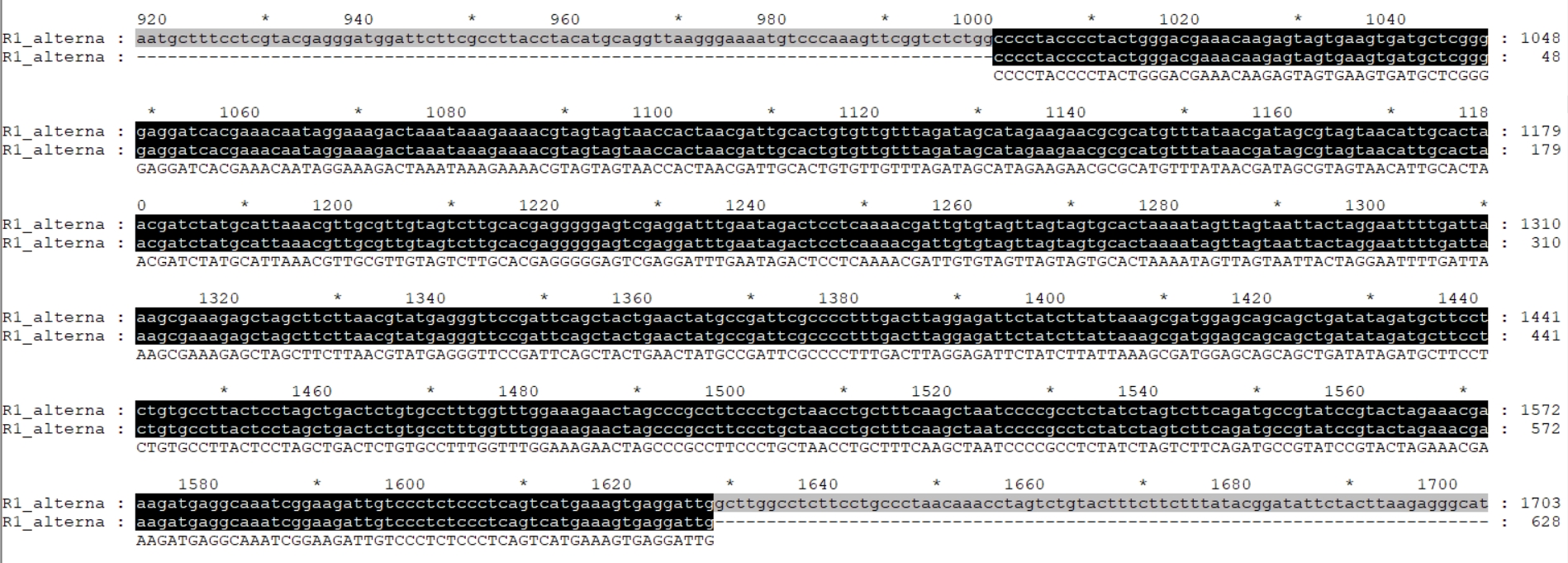


**Figure S7**. The results of Sanger sequencing for Junction 4 (f4/r4) of repeat sequence 1 (R1). The sequence at the top, in the middle and at the bottom are the assembled genome sequence, the sequence of the PCR products, and the consensus sequence.


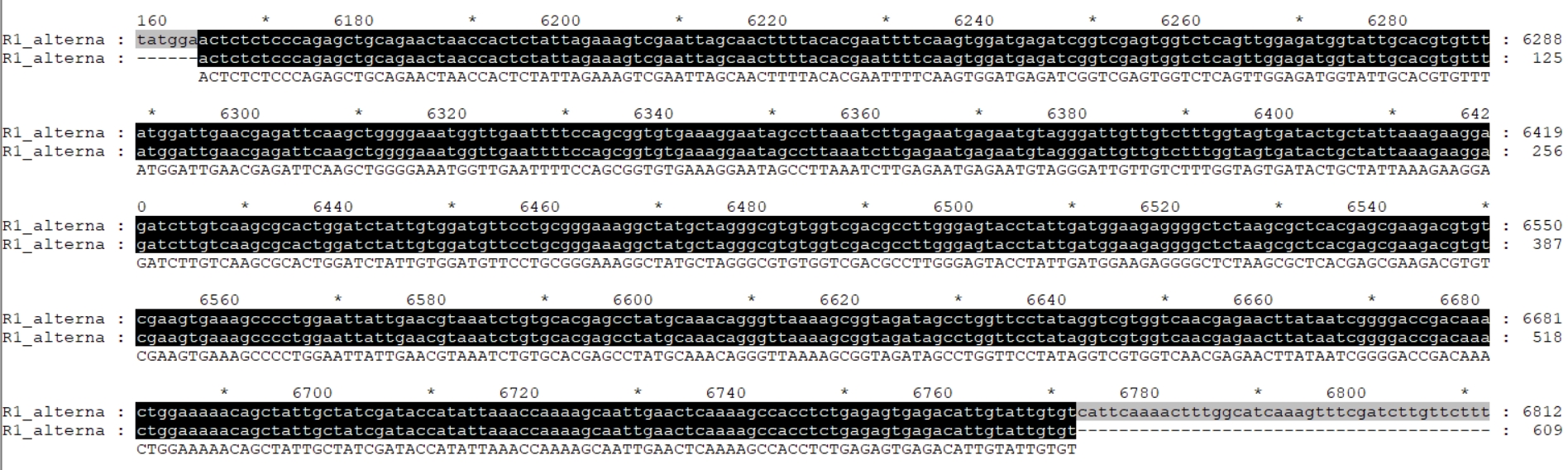


**Figure S8**. The results of Sanger sequencing for Junction 1 (f1/r1) of repeat sequence 3 (R3). The sequence at the top, in the middle and at the bottom are the assembled genome sequence, the sequence of the PCR products, and the consensus sequence.

**
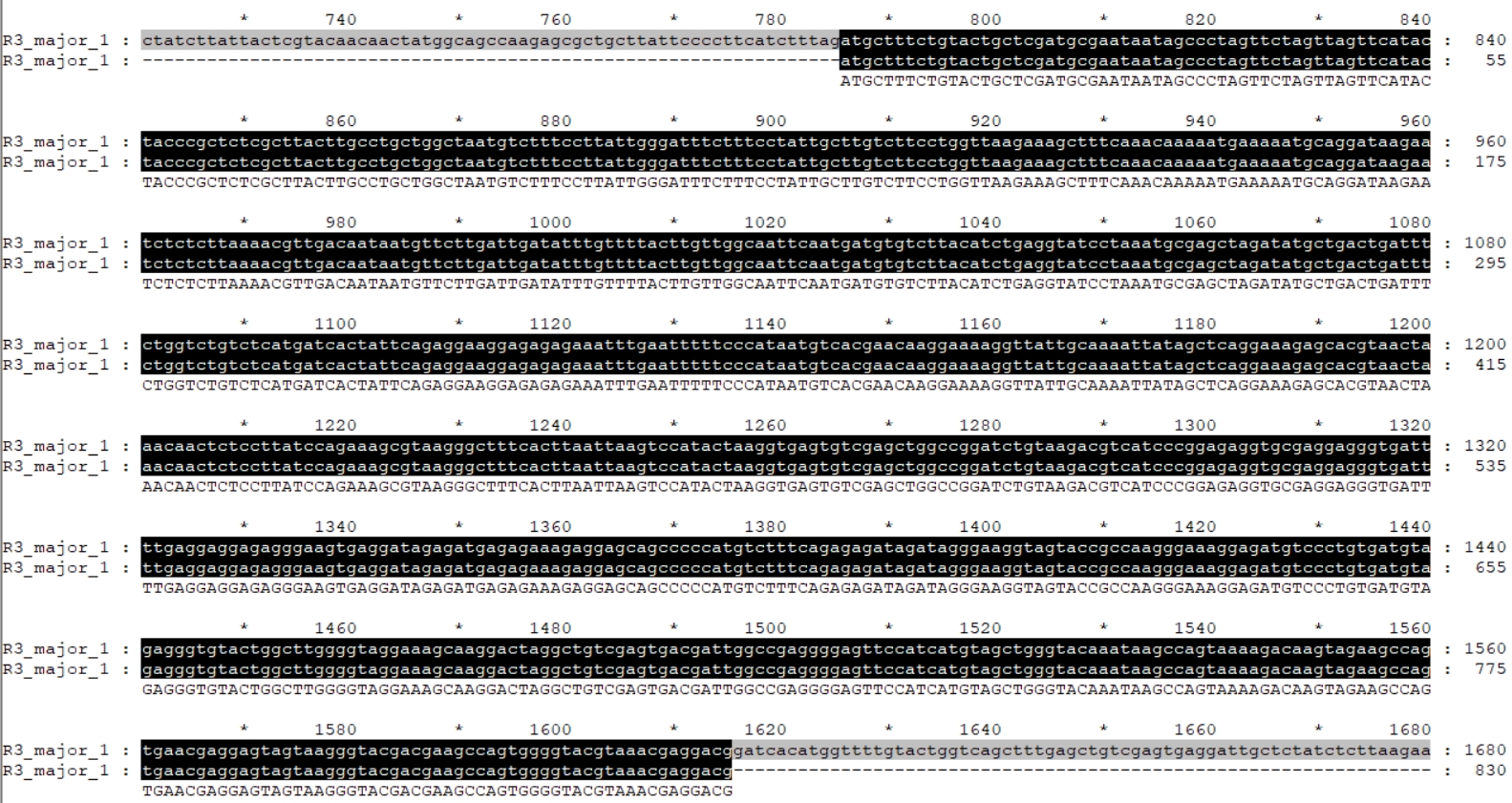
**

**Figure S9**. The results of Sanger sequencing for Junction 2 (f2/r2) of repeat sequence 3 (R3). The sequence at the top, in the middle and at the bottom are the assembled genome sequence, the sequence of the PCR products, and the consensus sequence.


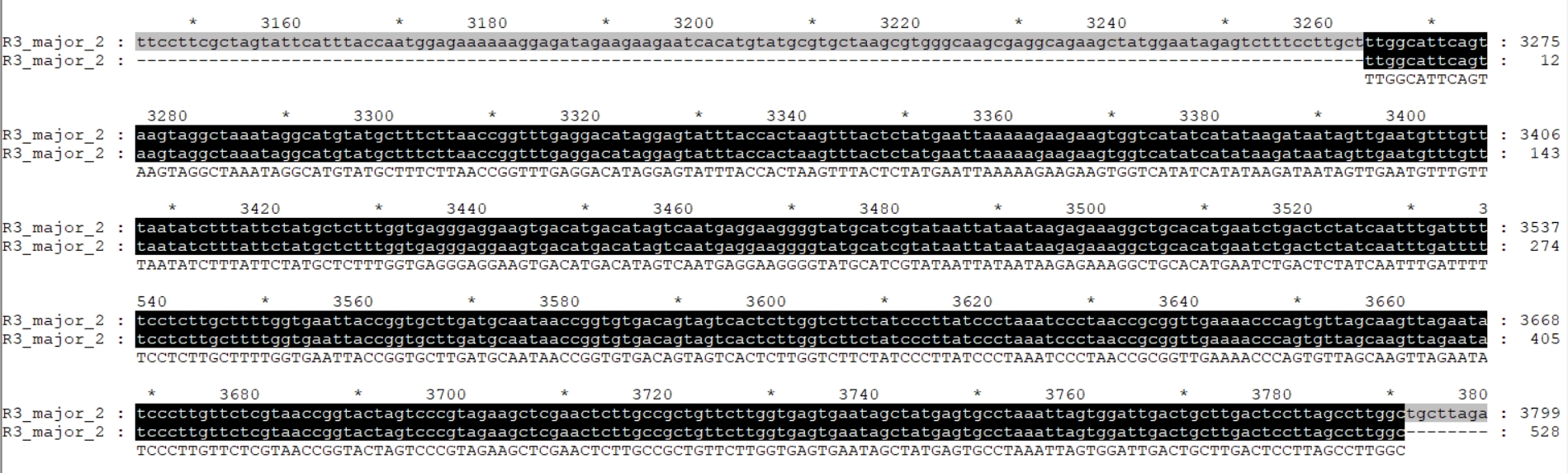


**Figure S10**. The results of Sanger sequencing for Junction 3 (f3/r3) of repeat sequence 3 (R3). The sequence at the top, in the middle and at the bottom are the assembled genome sequence, the sequence of the PCR products, and the consensus sequence.

**
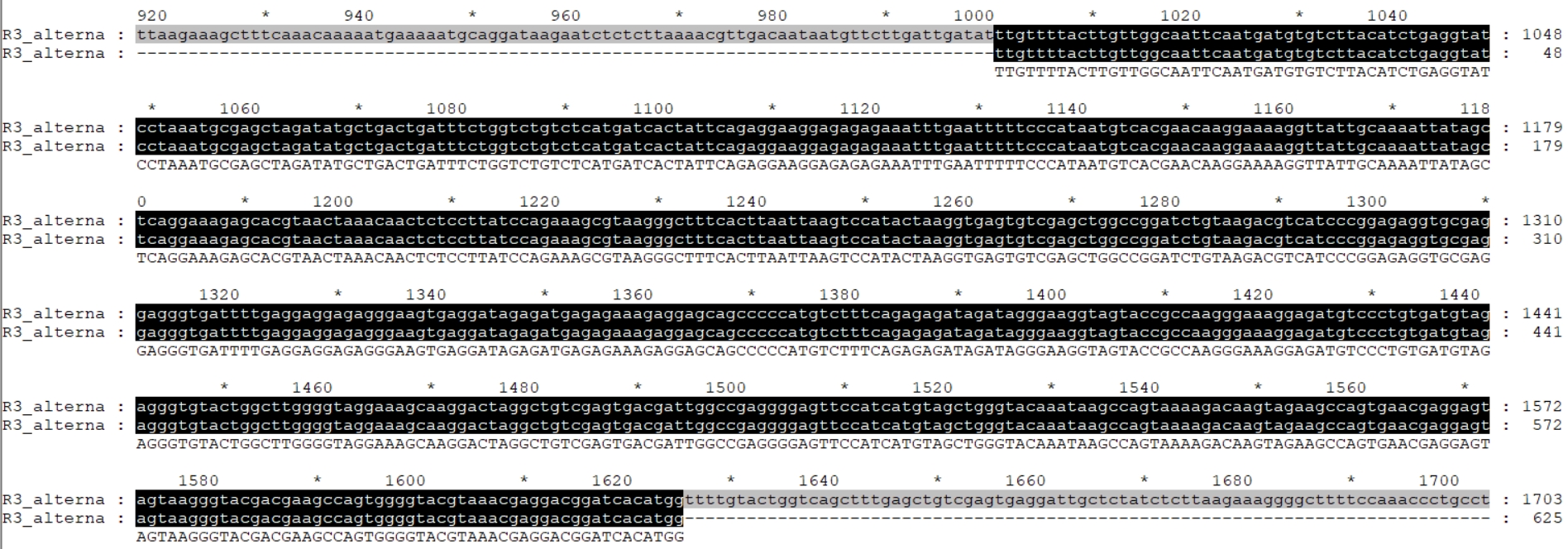
**

**Figure S11**. The results of Sanger sequencing for Junction 4 (f4/r4) of repeat sequence 3 (R3). The sequence at the top, in the middle and at the bottom are the assembled genome sequence, the sequence of the PCR products, and the consensus sequence.


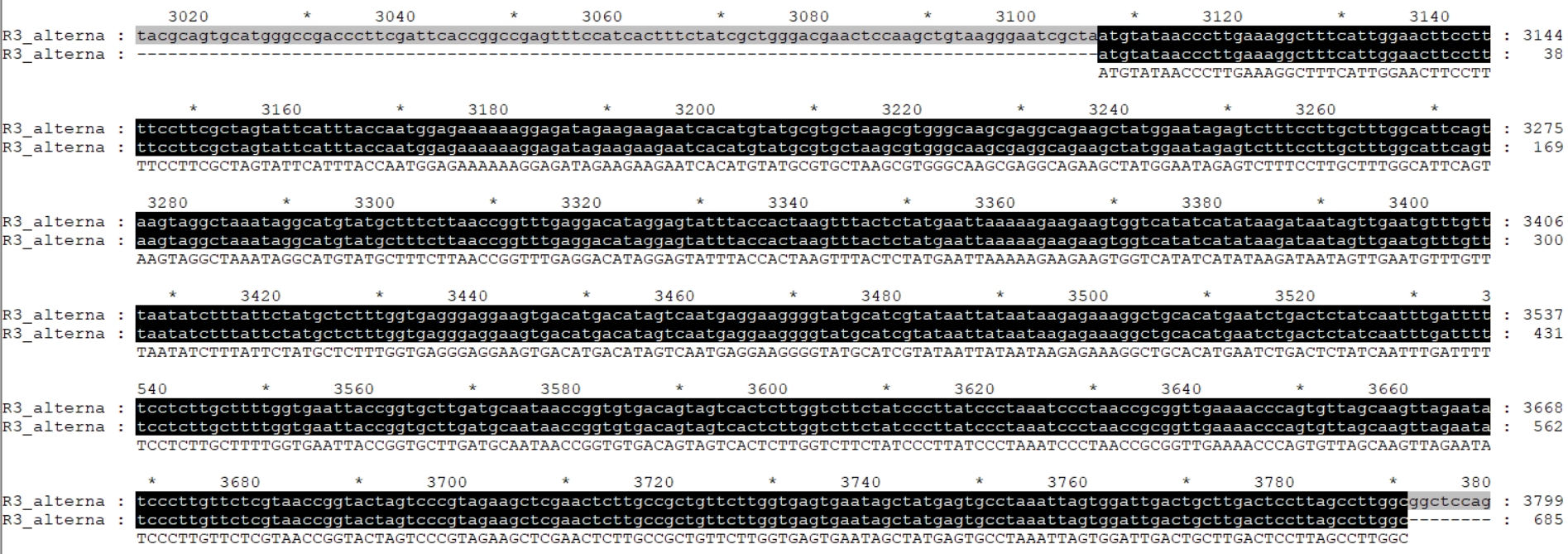


**Figure S12**. The results of Sanger sequencing for Junction 1 (f1/r1) of repeat sequence 77 (R77). The sequence at the top, in the middle and at the bottom are the assembled genome sequence, the sequence of the PCR products, and the consensus sequence.


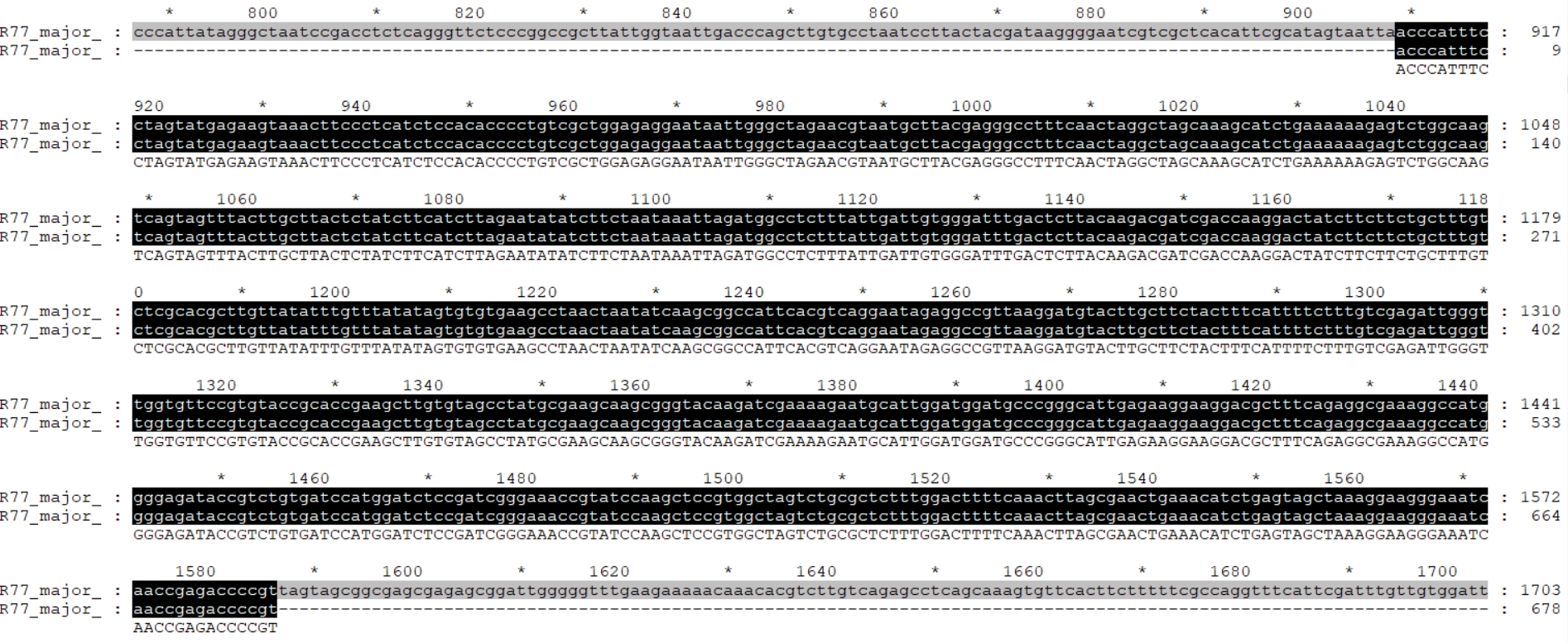


**Figure S13**. The results of Sanger sequencing for Junction 2 (f2/r2) of repeat sequence 77 (R77). The sequence at the top, in the middle and at the bottom are the assembled genome sequence, the sequence of the PCR products, and the consensus sequence.

**
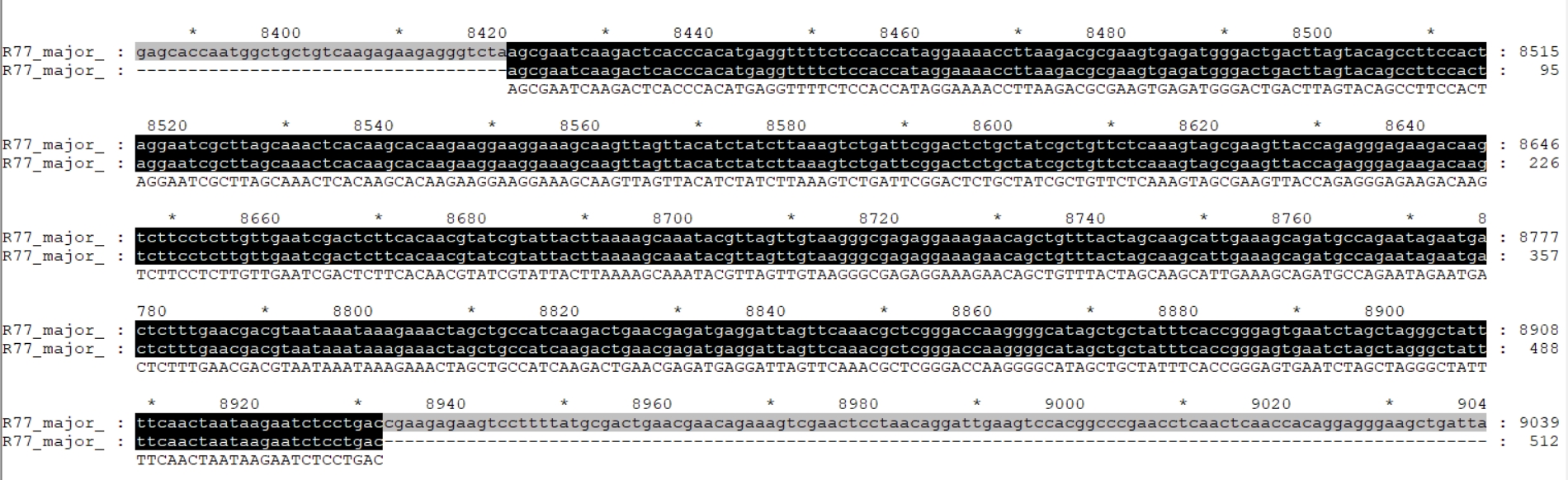
**

**Figure S14**. The results of Sanger sequencing for Junction 3 (f3/r3) of repeat sequence 77 (R77). The sequence at the top, in the middle and at the bottom are the assembled genome sequence, the sequence of the PCR products, and the consensus sequence.


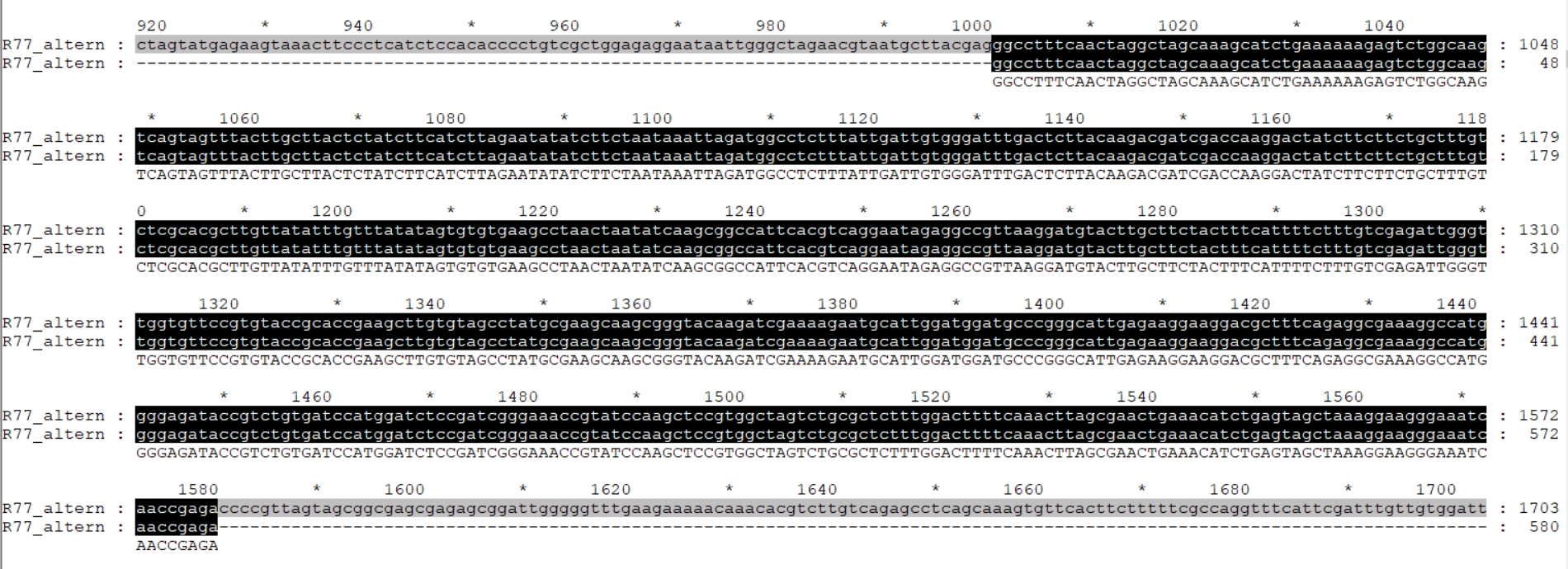


**Figure S15**. The results of Sanger sequencing for Junction 4 (f4/r4) of repeat sequence 77 (R77). The sequence at the top, in the middle and at the bottom are the assembled genome sequence, the sequence of the PCR products, and the consensus sequence.


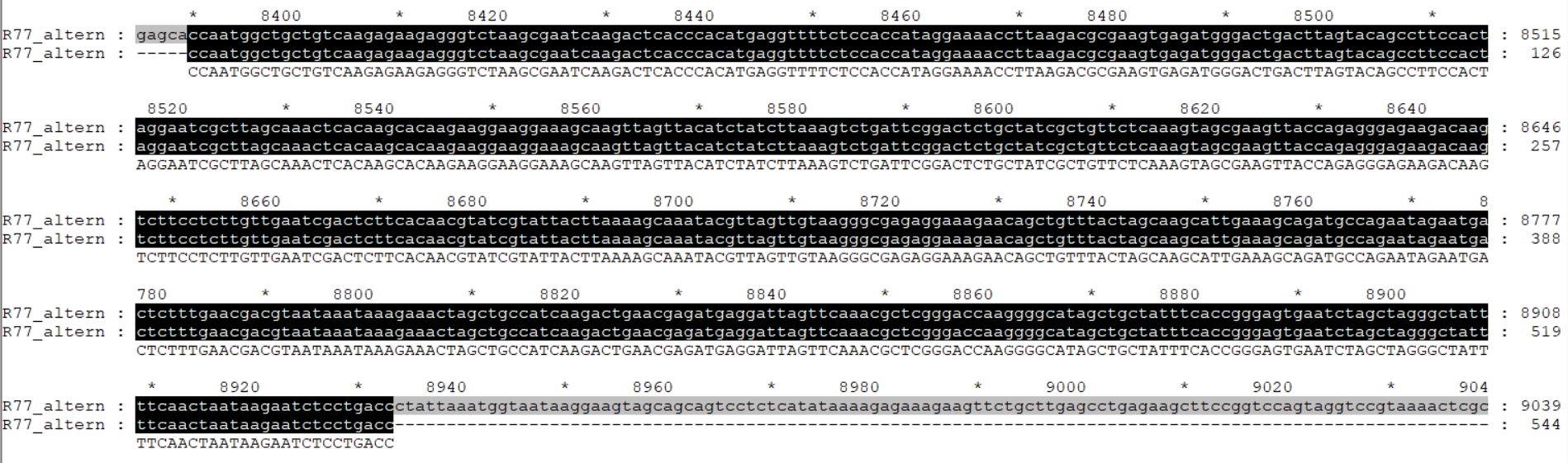


**Figure S16**. A schematic representation of the secondary configuration for the *R. glutinosa* mitogenome is provided. (A) A unitig graph for the *R. glutinosa* mitogenome was generated through de novo assembly of Illumina reads using Unicycler. This unitig graph consisted of seven contigs (depicted in yellow) that formed double bifurcating structures (DBSs) (B) The coverage depth of the Illumina short reads mapped to the *R. glutinosa* mitogenome sequences of the secondary configuration.


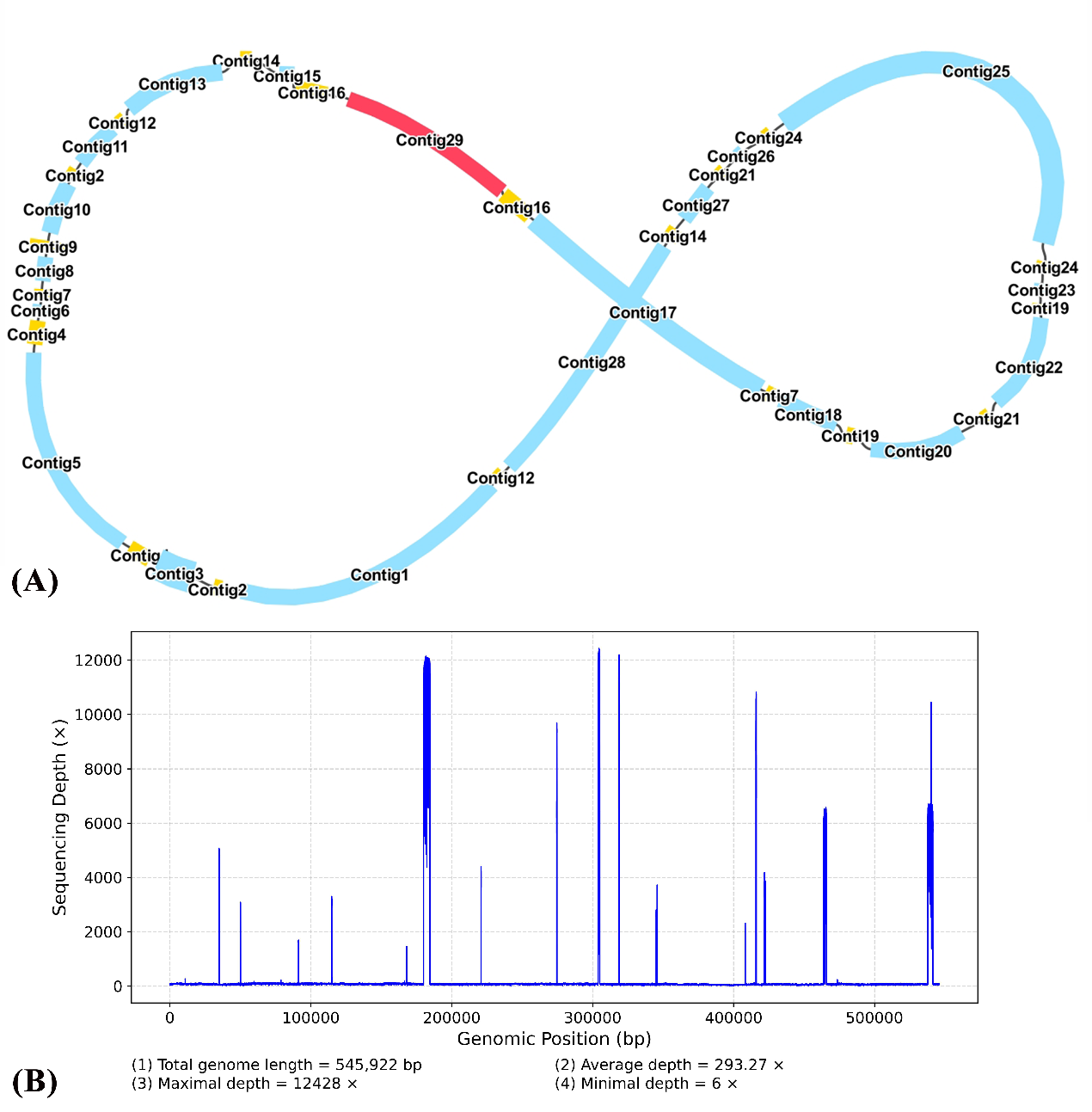


**Figure S17**. The panel A is a brid’s eye’s view of the RNA-seq reads aligned to the reference genes. The vertical lines show the nucleotides in the reads different from those in the reference. The read mapping result to the base level is shonw in panel B. The region shown in panel B is represented by a vertical red line in panel A. (A-B) The upsteam of chromosome2 reads mapping results. (C-D) The downsteam of chromosome2 reads mapping results.

**
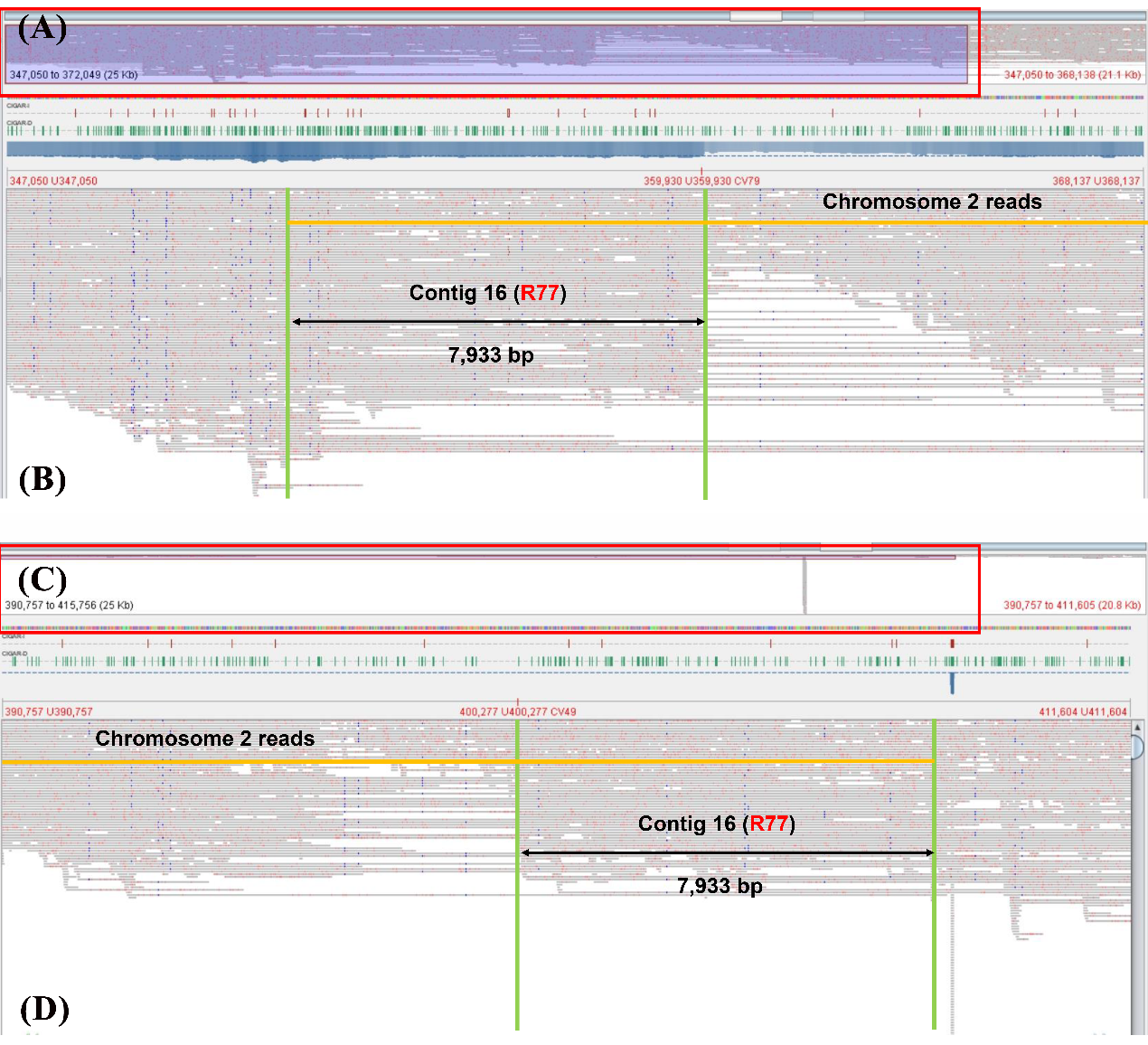
**

**Figure S18**. **Examplar homologos sequences bewteen the mitogenome and chloroplastome** (A) Aerial view of MTPT, and the red box represents the enlarged part. (B)Mapping of long reads onto MTPT3 on chromosome 1. The MTPT sequence is highlighted in a green box. The encompassed regions illustrate upstream (mitoDNA) - MTPT - downstream (mitoDNA) sequences. A mitochondrial read is highlighted in yellow, bordered by mitoDNA sequences with MTPT sequence in the middle.


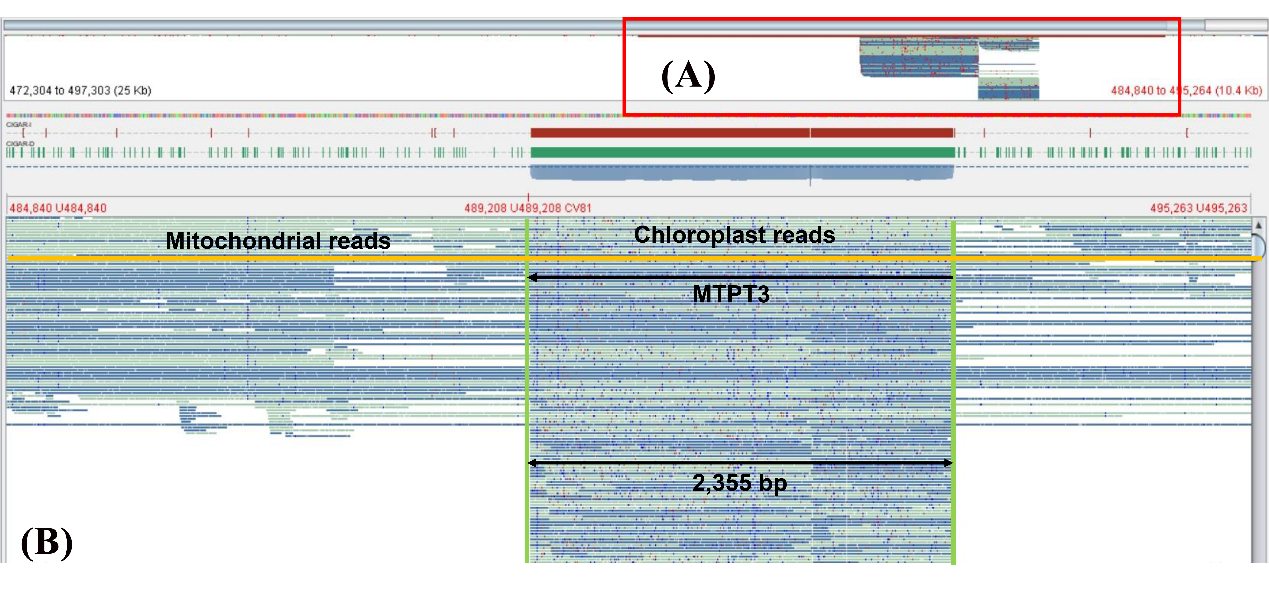


**Figure S19**. **Examplar homologos sequences bewteen the mitogenome and chloroplastome** (A) Aerial view of MTPT, and the red box represents the enlarged part. (B)Mapping of long reads onto MTPT7 on chromosome 1. The MTPT sequence is highlighted in a green box. The encompassed regions illustrate upstream (mitoDNA) - MTPT - downstream (mitoDNA) sequences. A mitochondrial read is highlighted in yellow, bordered by mitoDNA sequences with MTPT sequence in the middle.


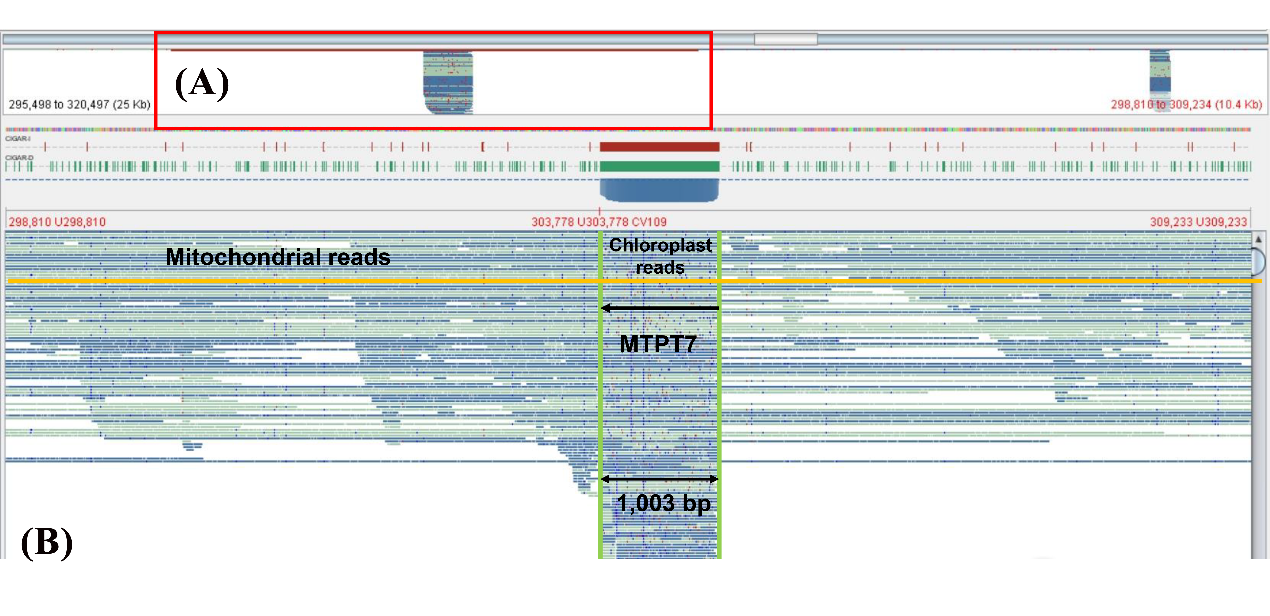


**Figure S20**. **Examplar homologos sequences bewteen the mitogenome and chloroplastome** (A) Aerial view of MTPT, and the red box represents the enlarged part. (B)Mapping of long reads onto MTPT14 on chromosome 1. The MTPT sequence is highlighted in a green box. The encompassed regions illustrate upstream (mitoDNA) - MTPT - downstream (mitoDNA) sequences. A mitochondrial read is highlighted in yellow, bordered by mitoDNA sequences with MTPT sequence in the middle.


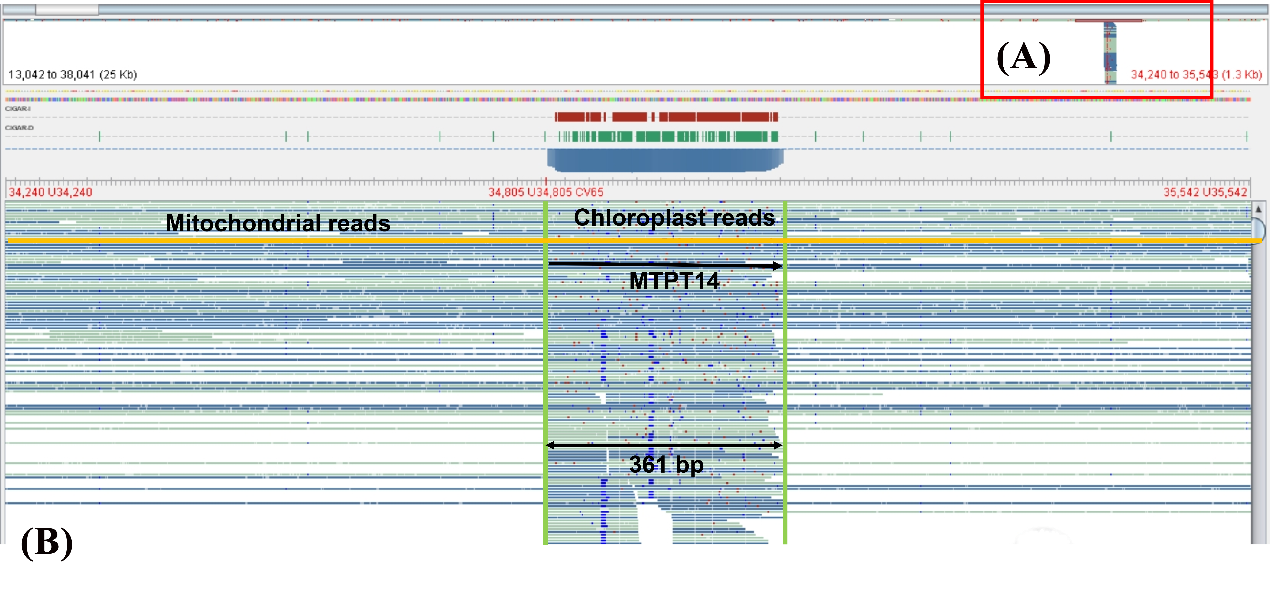


**Figure S21**. The stop codon of *atp6* gene (CAA→UAA, *Gln*→End) resulting from RNA editing. The panel A is a brid’s eye’s view of the RNA-seq reads aligned to the reference genes. The vertical lines show the nucleotides in the reads different from those in the reference. The read mapping result to the base level is shonw in panel B. The region shown in panel B is represented by a vertical red line in panel A.


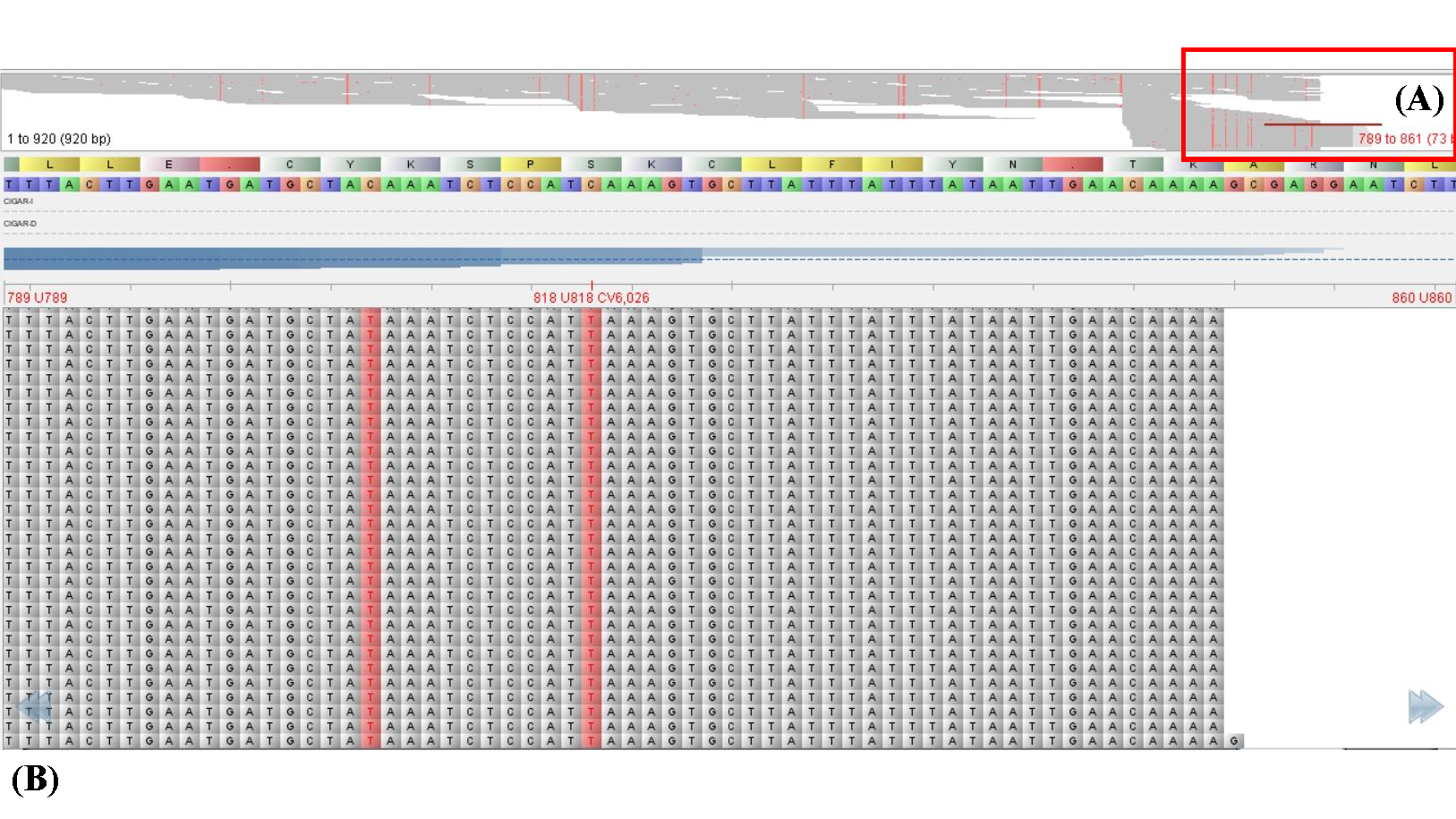


**Figure S22**. The stop codon of *rps10* gene (CGA→UGA, *Arg→*End) resulting from RNA editing. The panel A is a brid’s eye’s view of the RNA-seq reads aligned to the reference genes. The vertical lines show the nucleotides in the reads different from those in the reference. The read mapping result to the base level is shonw in panel B. The region shown in panel B is represented by a vertical red line in panel A.


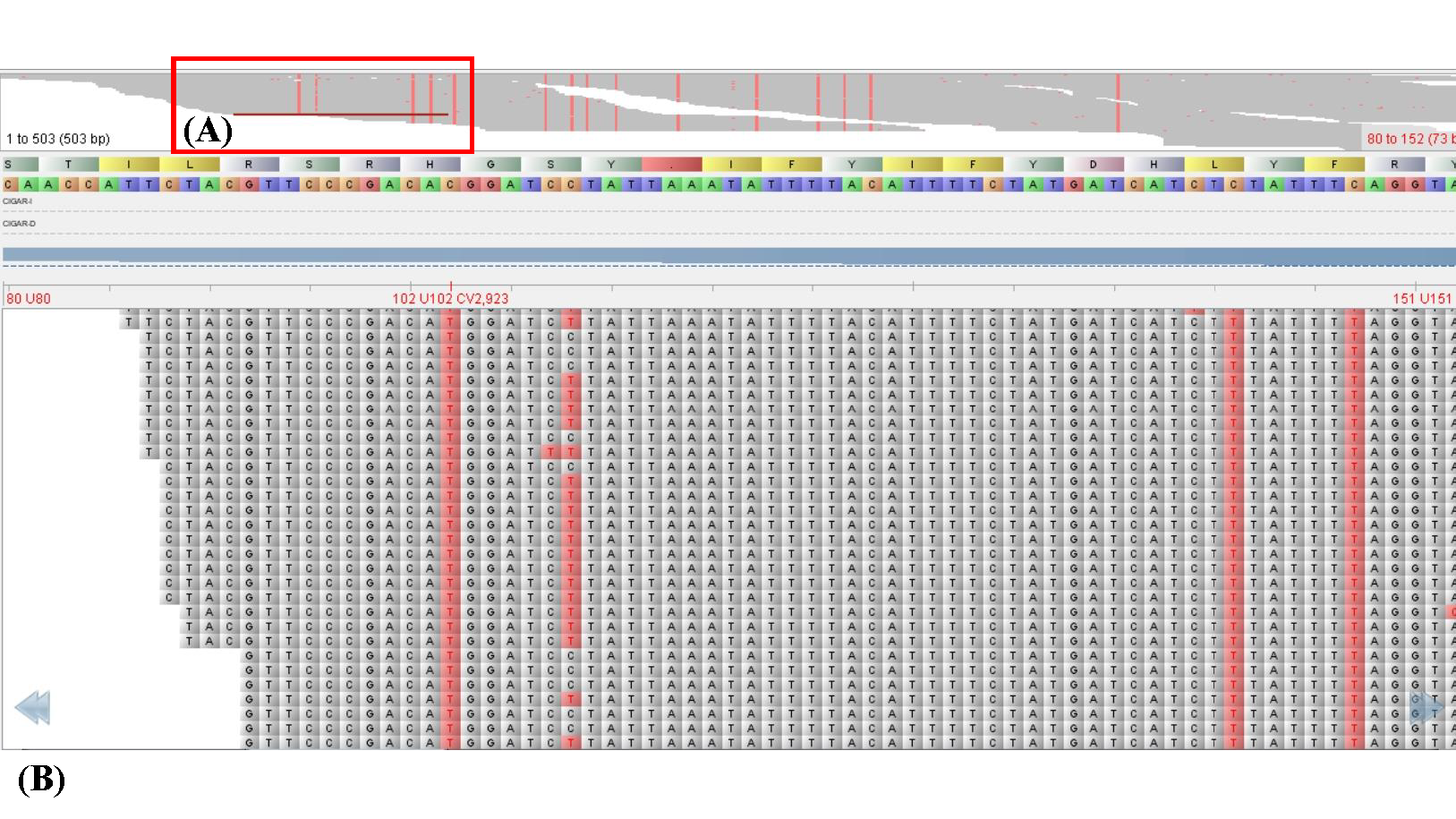


**Figure S23**. The start codon of *nad4L* gene (ACG→AUG, *Thr→Met*) resulting from RNA editing. The panel A is a brid’s eye’s view of the RNA-seq reads aligned to the reference genes. The vertical lines show the nucleotides in the reads different from those in the reference. The read mapping result to the base level is shonw in panel B. The region shown in panel B is represented by a vertical red line in panel A.


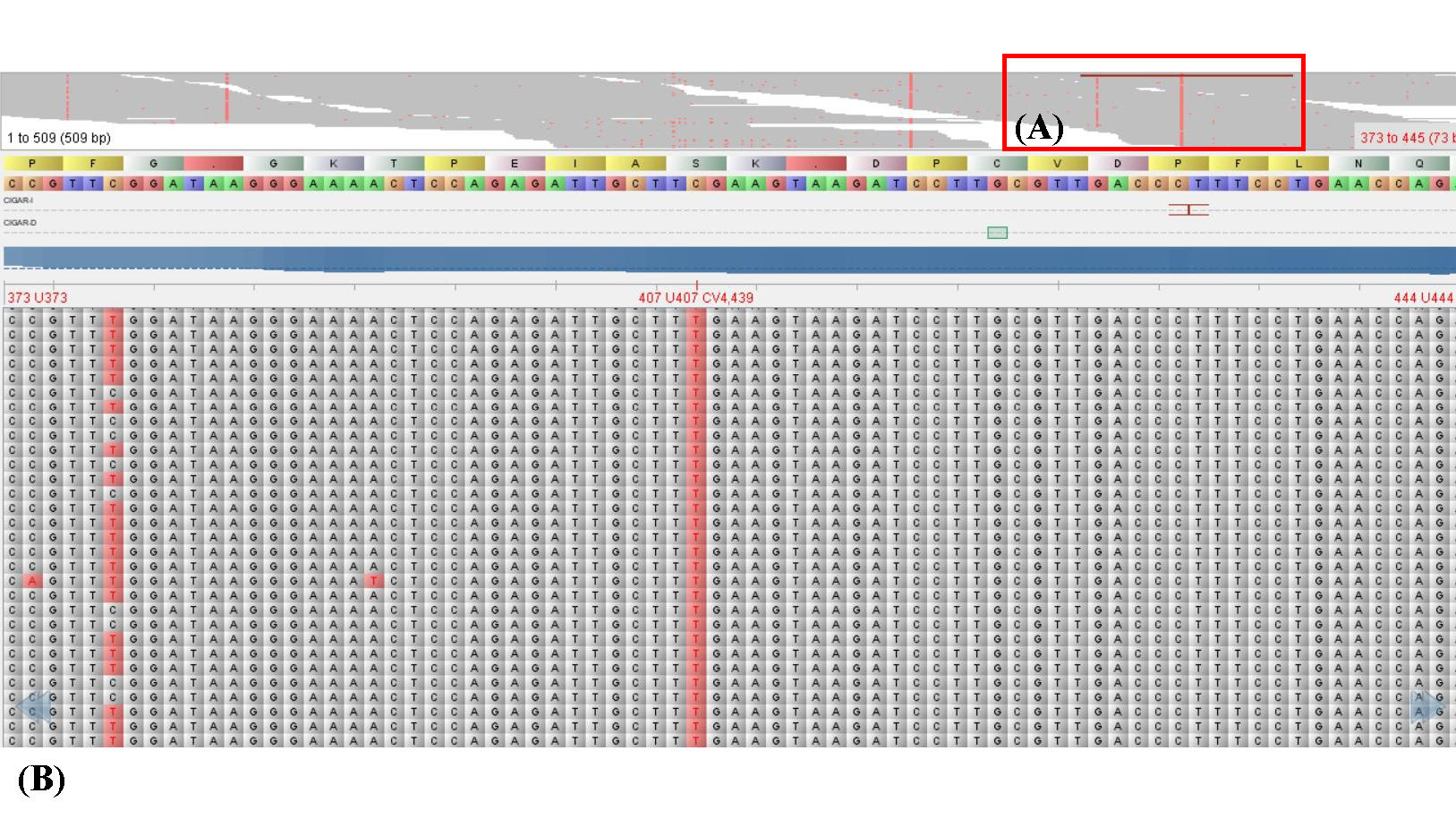

Supplement: Supplementary file 1 [file DataSheet_1.docx]
